# Transformation of Organostannanes Based on Photocleavage of C-Sn Bond via Single Electron Transfer Process

**DOI:** 10.1038/s41598-017-16806-3

**Published:** 2017-11-29

**Authors:** Han Li, Ruiwen Jin, Yawei Li, Aishun Ding, Xinqi Hao, Hao Guo

**Affiliations:** 1 Department of Chemistry, Fudan University, 220 Handan Road, Shanghai, 200433 People’s Republic of China; 2 College of Chemistry and Molecular Engineering, Zhengzhou University, No. 100 of Science Road, Henan, 450001 People’s Republic of China

## Abstract

In this work, we developed a new method for the transformation of organostannanes via radical process. In this reaction, highly reactive carbon radical species can be efficiently generated through HBr-catalyzed photocleavage of C-Sn bond via single electron transfer process. Under aerobic conditions, the *in situ* formed primary/secondary alkyl radicals can be further highly selectively oxidized into carboxylic acids/ketones, respectively.

## Introduction

**Figure 1:**
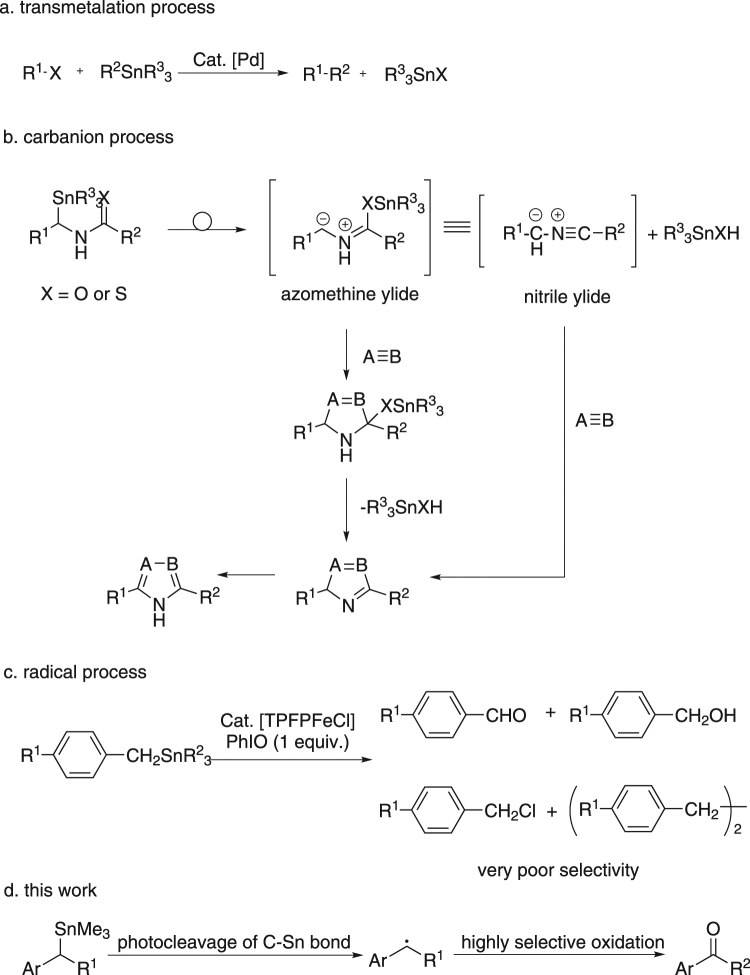
Transformation of organostannanes.

C-Sn bond is known as a kind of very important carbon-metal bond, which has numerous applications in organic chemistry^1,2^, biological chemistry^3–5^, and medicinal chemistry^6,7^. There are three main routes for the transformation of organostannanes, including transmetalation process^8–10^, carbanion process^11^, and radical process^12,13^. There are lots of reports on transmetalation process. For example, the Stille cross-coupling which is a powerful tool for the formation of C-C bond has been widely applied in synthetically organic chemistry (Fig. 1a)^8–10^. The C-Sn bond transformation via carbanion process has also been well studied. For instance, Komatsu *et al*. reported that a kind of aza five-membered heterocycles could be efficiently synthesized from *N*-(stannylmethyl)thioamides via a ylide key intermediate (Fig. 1b)^11^. However, achievements via radical process are rare. In 1995, Baciocchi *et al*. reported the Fe-catalyzed oxidation of benzyltrialkylstannanes by iodosylbenzene, which proceeded via a radical key intermediate. However, it showed very low value in synthetic chemistry, since only a complicated mixture could be afforded as the final product (Fig. 1c)^12^. Obviously, research in this field remained undeveloped. We wondered whether there is a new method that can efficiently generate highly reactive carbon radical intermediate through C-Sn bond cleavage, and more importantly, the subsequent transformation of the *in situ* generated carbon radical intermediate should be highly selective to make such a reaction synthetically useful. In our previous work, we reported the photoinduced HBr-catalyzed C-Si bond cleavage of benzylsilanes, in which benzyl radicals could be generated and highly selectively oxidized into benzoic acids^14^. In this reaction, the bromine radical generated from the pre-catalyst HBr could abstract a single electron from the C-Si σ bond, which would finally break the C-Si bond and form a benzyl radical. Considering the similarity of C-Sn and C-Si bond, we assumed that benzylstannane might also be converted into benzyl radical under similar reaction conditions. If it works, we will be able to develop a new method for the transformation of organostannanes via radical process. Herein, we wish to report our recent observation in this field (Fig. 1d).

## Results and Discussion

### Optimization and scope investigation

We conducted a series of reactions to screen the reaction conditions. Initially, the solvent effect was explored carefully. A solution of (4-methoxybenzyl)- trimethylstannane **1a** (0.2 mmol) and 20 mol% of HBr (aq., 48%) in different solvents (10 mL) was irradiated by a 300 W Xe lamp at rt under air atmosphere (entries 1–5, Table 1). A mixture of 4-methoxybenzaldehyde **2a** and 4-methoxybenzoic acid **3a** in a very poor selectivity was afforded when CH_3_CN or dimethyl carbonate (DMC) was used as the solvent (entries 1 and 2, Table 1). When dichloromethane (DCM) or acetone was used as the solvent, **3a** was formed as the major product in a better yield (entries 3 and 4, Table 1). To our delight, the utilization of ethyl acetate (EA) as the solvent delivered **3a** as the sole product in an 89% isolated yield (entry 5, Table 1). Thus, EA was chosen as the best solvent. Next, some bromide salts of group I elements, like LiBr, NaBr, or KBr, were tested, however, all the results were not satisfactory (entries 6–8, Table 1). Decreasing the amount of HBr to 10 mol% led to a diminished yield and a much lower chemoselectivity (entry 9, Table 1). When this reaction was run in the absence of light, no product was generated (entry 10, Table 1), which indicated that light played an important role in the reaction process. Thus, Condition A (**1a**

**Table 1:** Optimization of reaction conditions^a^.

Entry	Catalyst (mol%)	Solvent	Time (h)	Yield (%)^b^		
				1a	2a	3a

1	HBr (20)	CH_3_CN	24	8	52	15
2	HBr (20)	DMC	24	16	49	20
3	HBr (20)	DCM	24	0	2	59
4	HBr (20)	Acetone	24	0	3	75
5	HBr (20)	EA	6	0	0	94 (89)^c^
6^d^	LiBr•H_2_O (20)	EA	24	16	47	7
7^e^	NaBr (20)	EA	24	16	53	20
8^e^	KBr (20)	EA	24	0	6	69
9	HBr (10)	EA	24	14	38	24
10 ^f^	HBr (20)	EA	6	91	0	0

^a^A solution of **1a** (0.2 mmol) and catalyst in the tested solvent (10 mL) in a quartz reactor was irradiated by a 300 W Xe lamp at rt under air atomsphere. ^b^Yields were determined by ^1^H NMR analysis (400 MHz) of the crude reaction mixture employing CH_2_Br_2_ as the internal standard. ^c^Isolated yield of **3a**. ^d^H_2_O (3.8 μL) was added. ^e^H_2_O (4.5 μL) was added. ^f^The reaction was carried out without light.

(0.2 mmol), 20 mol% of HBr (aq., 48%), EA (10 mL), air (1 atm), 300 W Xe lamp, quartz, and rt) was chosen as the optimized reaction condition for the following studies.

With the optimized reaction conditions in hand, we investigated the substrate scope of this reaction with a series of benzyltrimethylstannane derivatives. As shown in Table 2, the reaction showed good tolerance. Substrates with electron-donating groups were found to be reactive, giving the corresponding products in good yields (**3a**–**e**). The substrate with no substitution on the phenyl ring, like benzyltrimethylstannane **1f**, was converted into **3 f** in an excellent yield. Nice yields were also obtained when electron-withdrawing groups were introduced. For substrate with a weak electron-withdrawing group, like chlorine or fluorine atom, the corresponding product was isolated in good yields (**3 g** and **3 h**). While the yields were a little lower, when the substitutions were changed into strong electron-withdrawing groups (**3i–l**). We were delight to see that naphthyl was also tolerant in this reaction, affording the corresponding 1- or 2-naphthoic acid, respectively (**3 m** and **3n**). Importantly, the benzoic acid derivatives **3** were formed as the sole product in all the above tests.

**Table 2:** Photooxidation of **1a–n** under Condition A^a^.


		
		
		
		
		

^a^A solution of **1** (0.2 mmol) and 20 mol% of HBr (aq., 48%) in EA (10 mL) in a quartz reactor was irradiated by a 300 W Xe lamp at rt under air atomosphere. The isolated yield was reported.

**Figure 2:**
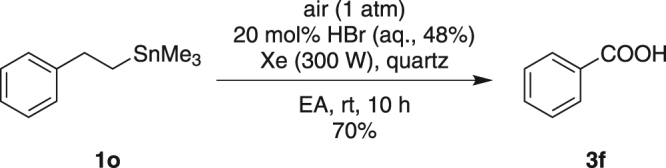
Photooxidation of **1o** under Condition A.

Interestingly, when trimethyl(phenethyl)stannane **1o** was applied under Condition A, only benzoic acid **3f** was formed in a good yield (Fig. 2). We assumed that phenylethyl radical was generated and it would be further oxidized into benzoic acid **3f** as the final product.

**Figure 3:**
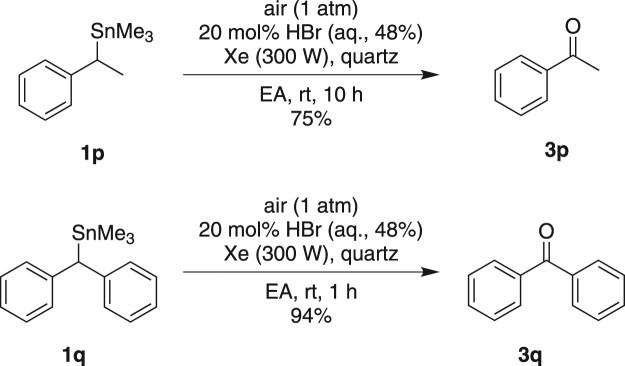
Photooxidation of **1p** and **1q** under Condition A.

In addition to the above primary alkyl stannanes, the reactivity of secondary alkyl stannanes were also examined. The corresponding ketones were formed as the sole product under Condition A (Fig. 3).

### Mechanism studies

In order to get a better understanding of this reaction, a series of control experiments were conducted to investigate the mechanism. As shown in Table 3, the photo reaction of **1a** proceeded smoothly to afford **3a** in a 94% NMR yield under Condition A (entry 1, Table 3). When the reaction was carried out without light, no reaction occurred and 91% of **1a** was recovered (entry 2, Table 3), which meant that light was the essential condition to start this reaction. When this reaction was run in the absence of air, 89% of **1a** was recovered without any other products isolated (entry 3, Table 3), which indicated that this reaction did not proceed without air. Next, the reaction was run for only 3 h (entry 4, Table 3). In this case, **1a** was not fully consumed. The corresponding benzaldehyde **2a** was formed. The desired benzoic acid **3a** was afforded in a low yield. This result might be a proof that benzaldehyde should be a key intermediate in the whole transformation. Then another reaction was run for 3 h under photo irradiation and a following 3 h without light (entry 5, Table 3). Almost the same result as the above test (entry 4, Table 3) was observed, which indicated that light played an important role not only in the initiation period, but also throughout the whole reaction process. Finally, the reaction was conducted without the catalyst HBr (entry 6, Table 3). The reaction speed was dramatically decreased with 46% of **1a** recovered. Only 9% of **3a** was yielded. Meanwhile, 46% of **2a** was formed. These results clearly showed that HBr not only facilitated the C-Sn bond cleavage at the beginning of reaction, but also benefited in the following oxidation step.

**Table 3:** Photo reaction of 1a under different conditions^a^.

Entry	HBr (20 mol%)	Air (1 atm)	hn (Xe lamp)	Time (h)	NMR yield (%)		
					1a	2a	3a

1	+	+	+	6	0	0	94%
2	+	+	−	6	91%	0	0
3	+	−	+	6	89%	0	0
4	+	+	+	3	46%	43%	10%
5	+	+	+^b^	3^c^ (6)^d^	47%	45%	8%
6	−	+	+	6	46%	46%	9%

^a^A solution of **1a** (0.2 mmol) in EA (10 mL) in a quartz reactor was irradiated by a 300 W Xe lamp at rt under air atmosphere. ^b^The photo irradiation was stopped after 3 h. ^c^Reaction time under photo irradiation. ^d^Total reaction time.

**Figure 4:**
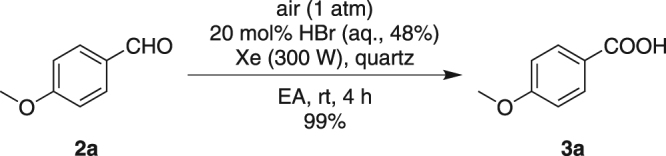
Photo reaction of **2a** under Condition A.

To further prove that benzaldehyde was the intermediate of this whole transformation, **2a** was directly employed under Condition A. After 4 h, **3a** was afforded in a 99% yield, which fully supported this inference (Fig. 4).

**Figure 5:**
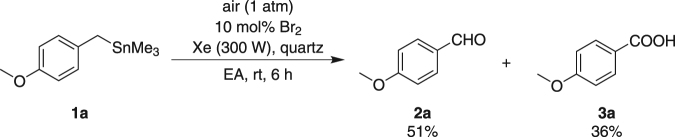
Br_2_-catalyzed photo reaction of **1a**.

According to our previous work^14^, Br_2_ was formed under similar photo reaction conditions and played a key role in the catalytic process. Thus, 10 mol% of Br_2_ was employed instead of HBr to identify its catalytic property in this reaction. As a result, **2a** was formed in a 51% yield and **3a** was afforded in a 36% yield after irradiated for 6 h (Fig. 5), which indicated that Br_2_ could also catalyze this reaction with relatively lower efficiency.

**Figure 6:**
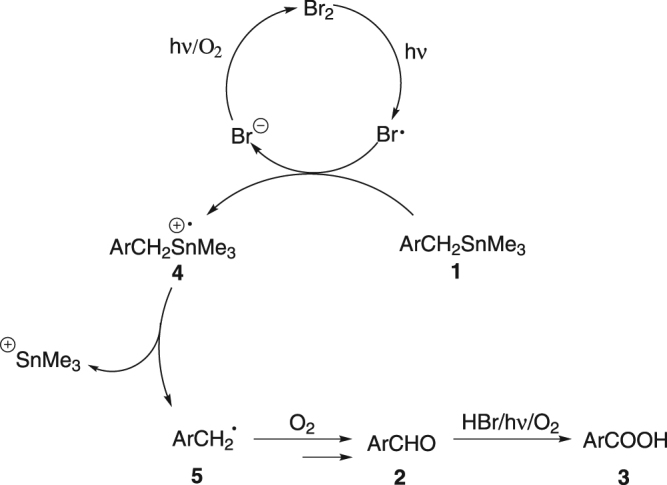
Proposed mechanism.

Based on the above experiment results and literature precedents^14–20^, a possible mechanism was proposed as shown in Fig. 6. Firstly, Br^−^ was oxidized into Br_2_ by oxygen under photo irradiation^14^. Br_2_ was then photochemically converted into bromine radical^15,16,21,22^. In the presence of HBr, bromine radical had a relatively high oxidation potential^18,23^ which was strong enough to capture one electron from C-Sn σ bond of **1**. Thus, the transformation of organostannanes was initiated. After being grabbed one electron from the C-Sn bond, the highly unstable radical cation **4** was formed. The subsequent heterolytic cleavage of C-Sn bond in **4** proceeded quickly to give carbon radical **5**. It could be easily oxidized into benzaldehyde **2** ^24–26^, and further oxidized into the final product benzoic acid **3** ^27^ under Condition A.

## Conclusions

In summary, we have developed a novel method for the transformation of organostannanes via radical process. Under photo irradiation, HBr could efficiently catalyze the heterolytic cleavage of C-Sn bond in organostannanes and the subsequent highly selective oxidation. The mechanism was well studied. In this reaction, bromine acted as a medium of photochemical single electron transfer process. Such a catalyst is simple, effective, cheap, and easy to handle. Further studies in this field is in progress in our laboratory.

## Methods

### Synthesis of 4-methoxybenzoic acid (3a)

**1a** (57 mg, 0.20 mmol), EA (10 mL), and HBr (aq., 48%) (4.5 μL, 0.04 mmol) were added to a quartz reaction flask which was equipped with a magnetic stirrer and a condenser. The mixture was irradiated by a Xe lamp (300 W) at rt in the open air. The photoreaction was completed after 6 hours as monitored by TLC (eluent: petroleum ether: ethyl acetate = 10:1). The solvent was removed and the residue was purified by flash chromatography on silica gel (eluent: petroleum ether: ethyl acetate = 3:1) to afford **3a** as a solid (27 mg, 89%); ^1^H NMR (400 MHz, CDCl_3_) *δ* 8.07 (d, *J* = 8.4 Hz, 2 H), 6.95 (d, *J* = 8.4 Hz, 2 H), 3.88 (s, 3 H).

## Electronic supplementary material

Supplementary Information
