# Supplementary material for: Transformation of Organostannanes Based on Photocleavage of C-Sn Bond via Single Electron Transfer Process
Source: Sci Rep. 2017 Nov 29;7:16559. doi: 10.1038/s41598-017-16806-3 (PMC5707384; doi:10.1038/s41598-017-16806-3)
Supplement: Supplementary file 1 — Supplementary Information [file 41598_2017_16806_MOESM1_ESM.pdf]

Supplementary Information

Transformation of Organostannanes Based on Photocleavage of C-Sn Bond via Single Electron Transfer Process

Han Li,[2,#] *Ruiwen Jin,*[1,#] *Yawei Li,*[1] *Aishun Ding,*[1] *Xinqi Hao,*[2,*] *Hao Guo*[1,*]

[1] Department of Chemistry, Fudan University, 220 Handan Road, Shanghai 200433, People's Republic of China

[2] College of Chemistry and Molecular Engineering, Zhengzhou University, No. 100 of Science Road, Henan, 450001, People's Republic of China

[#] Both authors contributed equally to this work.

* To whom correspondence should be addressed, E-mail: Hao_Guo@fudan.edu.cn (Tel: +86-21-55664361, Fax: +86-21-55664361); xqhao@zzu.edu.cn (Tel: +86-371-67763866).

Experimental Section	S2
NMR Spectra	S17
References	S41

Experimental Section

General experimental methods

All reactions were carried out using a PLS-SXE300UV reactor with Xe lamp (300 W) as the irradiation source. ^{1}H (400 MHz), ^{13}C (100 MHz), and ^{19}F (376 MHz) NMR spectra of samples in $CDCl_3$ (unless stated otherwise) were recorded on an AVANCE III 400 spectrometer. IR spectra were recorded on a Avatar 360 FT-IR spectrometer. HRMS (EI) determinations were carried out on a Water GCT CA176 spectrometer.

Synthesis of (2-methoxybenzyl)trimethylstannane (1c)[1]

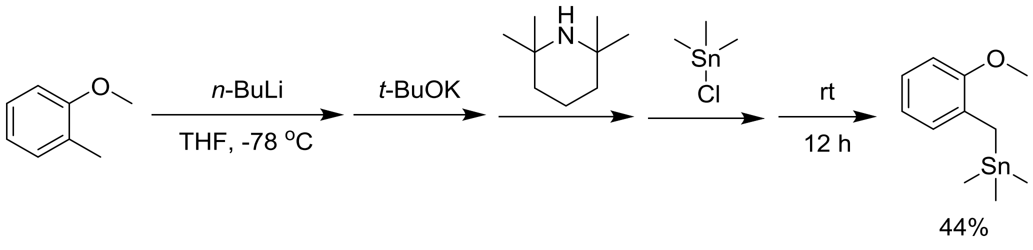

1c

Under argon atmosphere, 2-methylanisole (375 μL, 3.0 mmol) and anhydrous THF (15 mL) were added into a dry 100 mL of three-necked flask. The mixture was stirred at -78 °C for 5 min. Then *n*-BuLi (2.4 M in hexane) (1.7 mL, 4.1 mmol) was added dropwise. After stirred at -78 °C for 10 min, a solution of *t*-BuOK (511 mg, 4.2 mmol) in anhydrous THF (8 mL) and 2,2,6,6-tetramethylpiperidine (510 μL, 3.0 mmol) were added dropwise. The mixture was stirred at -78 °C for 20 min. Then trimethyltin chloride (713 mg, 3.6 mmol) was added, after which the reaction mixture was allowed to warm to rt. After stirred at rt for 12 hours, the reaction was quenched by saturated solution of NH_4Cl (10 mL). The mixture was extracted with ethyl acetate (20 mL x 3). The combined organic layer was dried over $MgSO_4$. Filtration, concentration, and purification by flash chromatography on silica gel (eluent: petroleum ether) afforded **1c** as a liquid (377 mg, 44%); ^{1}H NMR (400 MHz, $CDCl_3$) δ 7.07-6.99 (m, 2 H), 6.87-6.75 (m, 2 H), 3.80 (s, 3 H), 2.26 (s, 2 H), 0.02 (s, 9 H).

Synthesis of (1-(phenyl)ethyl)trimethylstannane (1p)

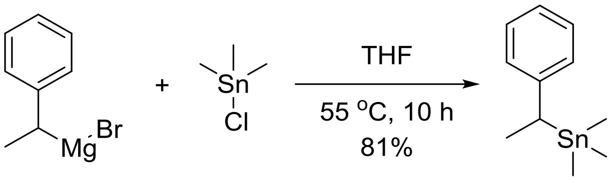

1p

Under argon atmosphere, Mg (328 mg, 13.5 mmol), anhydrous THF (15 mL), and trimethyltin chloride (562 mg, 2.8 mmol) were added into a dry 100 mL of three-necked flask. The mixture was refluxed for 15 min, after which a drop of I_2 was added. Then a solution of (1-bromoethyl)benzene (820 μL, 6.0 mmol) in anhydrous THF (15 mL) was added dropwise. After stirred at 55 °C for 10 hours, the reaction was quenched by saturated solution of NH_4Cl (10 mL). The mixture was extracted with ethyl acetate (30 mL x 3). The combined organic layer was dried over $MgSO_4$. Filtration, concentration, and purification by flash chromatography on silica gel (eluent: petroleum ether) afforded **1p** as a liquid (611 mg, 80%); ^{1}H NMR (400 MHz, $CDCl_3$) δ 7.44-7.36 (m, 2 H), 7.23-7.14 (m, 3 H), 2.82 (q, J = 7.6 Hz, 1 H), 1.72 (d, J = 7.6 Hz, 3 H), 0.16 (s, 9 H); ^{13}C NMR (100 MHz, $CDCl_3$) δ 148.3, 128.2, 125.3, 123.4, 27.0, 16.6, -11.0; IR (neat) 1601, 1492, 1450 cm^{-1}; HRMS (EI) calcd for $C_{11}H_{18}{}^{112}Sn$ 262.0457, found 262.0455.

Synthesis of (diphenylmethyl)trimethylstannane (1q)

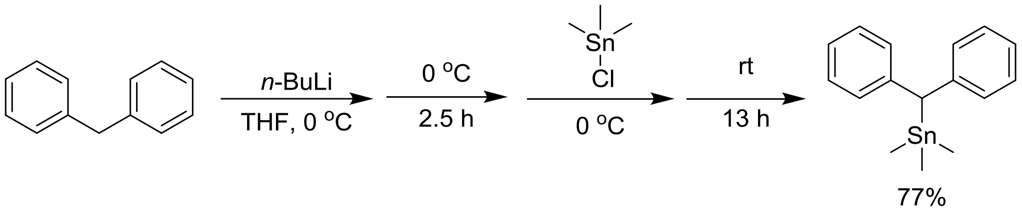

1q

Under argon atmosphere, diphenylmethane (0.5 mL, 3.0 mmol) and anhydrous THF (25 mL) were added into a dry 100 mL of Schlenk flask. The mixture was stirred at 0 °C for 5 min. Then *n*-BuLi (2.5 M in hexane) (2.5 mL, 6.3 mmol) was added dropwise. After stirred at 0 °C for 2.5 hours, trimethyltin chloride (1.132 g, 5.7 mmol) was added at 0 °C. After stirred at rt for 13 hours, the reaction was quenched by saturated solution of NH_4Cl (10 mL). The mixture was extracted with ethyl acetate (20 mL x 3). The combined organic layer was dried over $MgSO_4$. Filtration, concentration, and

purification by flash chromatography on silica gel (eluent: petroleum ether) afforded **1q** as a liquid (763 mg, 77%); ^{1}H NMR (400 MHz, $CDCl_3$) δ 7.32-7.24 (m, 4 H), 7.18 (d, J = 7.2 Hz, 4 H), 7.11 (t, m, 2 H), 4.02 (s, 1 H), 0.08 (s, 9 H); ^{13}C NMR (100 MHz, $CDCl_3$) δ 144.1, 128.5, 127.8, 124.5, 42.9, -8.9; IR (neat) 1596, 1491, 1447 cm^{-1}; HRMS (EI) calcd for $C_{16}H_{20}{}^{112}Sn$ 324.0613, found 324.0615.

Typical Procedure I for the synthesis of benzyltrimethylstannanes.

Synthesis of (4-methoxybenzyl)trimethylstannane (1a)

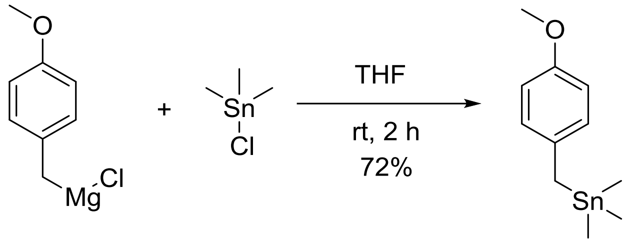

1a

Under argon atmosphere, Mg (251 mg, 10.5 mmol), anhydrous THF (20 mL), trimethyltin chloride (1.012 g, 5.1 mmol), and a drop of I_2 were added into a dry 100 mL of three-necked flask. The mixture was stirred at rt for 15 min. Then a solution of 4-methoxybenzyl chloride (560 μL, 5.5 mmol) in anhydrous THF (5 mL) was added dropwise. After stirred at rt for 2 hours, the reaction was quenched by saturated solution of NH_4Cl (10 mL). The mixture was extracted with ethyl acetate (20 mL x 3). The combined organic layer was dried over $MgSO_4$. Filtration, concentration, and purification by flash chromatography on silica gel (eluent: petroleum ether) afforded **1a** as a liquid (1.044 g, 72%); ^{1}H NMR (400 MHz, $CDCl_3$) δ 6.92 (d, J = 8.6 Hz, 2 H), 6.78 (d, J = 8.6 Hz, 2 H), 3.78 (s, 3 H), 2.27 (s, 2 H), 0.06 (s, 9 H); ^{13}C NMR (100 MHz, $CDCl_3$) δ 155.9, 134.9, 127.6, 113.8, 55.2, 18.7, -10.2; IR (neat) 1609, 1576, 1506, 1463, 1438 cm^{-1}; HRMS (EI) calcd for $C_{11}H_{18}O^{112}Sn$ 278.0406, found 278.0399.

The following compounds were prepared according to Typical Procedure I.

(1) (3-Methoxybenzyl)trimethylstannane (1b)

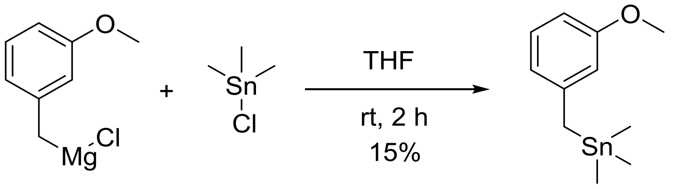

1b

The reaction of 3-methoxybenzyl chloride (440 μL, 3.0 mmol), Mg (153 mg, 6.3 mmol), anhydrous THF (20 mL), trimethyltin chloride (597 mg, 3.0 mmol), and a drop of I_2 afforded **1b** as a liquid (125 mg, 15%); ^{1}H NMR (400 MHz, $CDCl_3$) δ 7.12 (t, J = 7.8 Hz, 1 H), 6.63-6.54 (m, 3 H), 3.79 (s, 3 H), 2.32 (s, 2 H), 0.08 (s, 9 H); ^{13}C NMR (100 MHz, $CDCl_3$) δ 159.7, 144.9, 129.2, 119.4, 112.4, 108.5, 55.0, 20.4, -10.0; IR (neat) 1592, 1580, 1485, 1467, 1455, 1430 cm^{-1}; HRMS (EI) calcd for $C_{11}H_{18}O^{112}Sn$ 278.0406, found 278.0409.

(2) (4-(*Tert*-butyl)benzyl)trimethylstannane (1d)

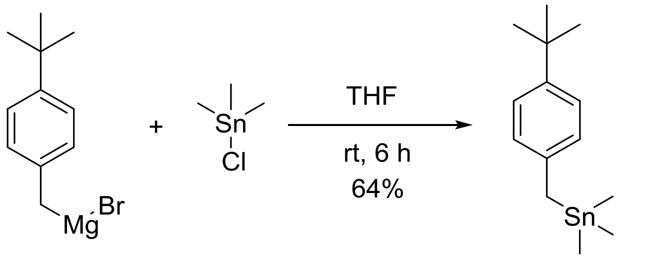

1d

The reaction of 4-(*tert*-butyl)benzyl bromide (1.1 mL, 6.0 mmol), Mg (195 mg, 8.0 mmol), anhydrous THF (15 mL), trimethyltin chloride (593 mg, 3.0 mmol), and a drop of I_2 afforded **1d** as a liquid (590 mg, 64%); ^{1}H NMR (400 MHz, $CDCl_3$) δ 7.19 (d, J = 8.0 Hz, 2 H), 6.90 (d, J = 8.0 Hz, 2 H), 2.27 (s, 2 H), 1.28 (s, 9 H), 0.04 (s, 9 H); ^{13}C NMR (100 MHz, $CDCl_3$) δ 145.8, 139.7, 126.5, 125.1, 34.1, 31.4, 19.4, -10.0; IR (neat) 1609, 1507, 1477, 1464 cm^{-1}; HRMS (EI) calcd for $C_{14}H_{24}{}^{112}Sn$ 304.0926, found 304.0930.

(3) (4-(Phenyl)benzyl)trimethylstannane (1e)

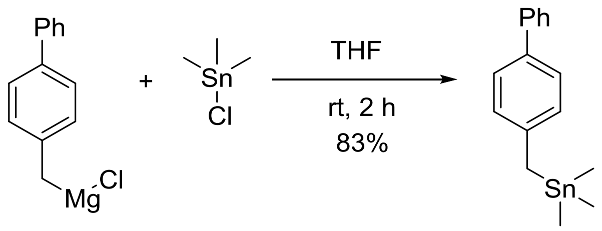

1e

The reaction of 4-(phenyl)benzyl chloride (840 mg, 4.1 mmol), Mg (180 mg, 7.4 mmol), anhydrous THF (25 mL), trimethyltin chloride (610 mg, 3.1 mmol), and a drop of I_2 afforded **1e** as a liquid (839 mg, 83%); ^{1}H NMR (400 MHz, $CDCl_3$) δ 7.61 (d, J = 7.6 Hz, 2 H), 7.50-7.40 (m, 4 H), 7.36-7.28 (m, 1 H), 7.08 (d, J = 8.0 Hz, 2 H), 2.38 (s, 2 H), 0.11 (s, 9 H); ^{13}C NMR (100 MHz, $CDCl_3$) δ 142.4, 141.1, 135.9, 128.6, 127.2, 127.1, 126.9, 126.6, 19.8, -10.0; IR (neat) 1609, 1523, 1484, 1447, 1410 cm^{-1}; HRMS (EI) calcd for $C_{16}H_{20}{}^{112}Sn$ 324.0613, found 324.0617.

(4) Benzyltrimethylstannane (1f)

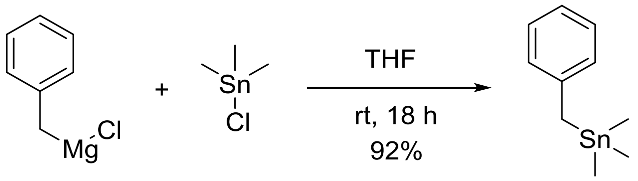

1f

The reaction of benzyl chloride (1.0 mL, 8.7 mmol), Mg (357 mg, 14.7 mmol), anhydrous THF (20 mL), trimethyltin chloride (1.191 g, 6.0 mmol), and a drop of I_2 afforded **1f** as a liquid (1.408 g, 92%); ^{1}H NMR (400 MHz, $CDCl_3$) δ 7.26-7.18 (m, 2 H), 7.06-6.99 (m, 3 H), 2.35 (s, 2 H), 0.09 (s, 9 H); ^{13}C NMR (100 MHz, $CDCl_3$) δ 143.1, 128.3, 126.8, 123.1, 20.2, -10.1; IR (neat) 1600, 1489, 1449 cm^{-1}; HRMS (EI) calcd for $C_{10}H_{16}{}^{112}Sn$ 248.0300, found 248.0304.

(5) (4-Chlorobenzyl)trimethylstannane (1g)

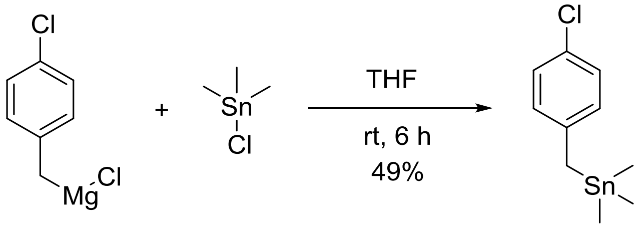

1g

The reaction of 4-chlorobenzyl chloride (0.5 mL, 4.0 mmol), Mg (145 mg, 6.0 mmol),

anhydrous THF (25 mL), trimethyltin chloride (593 mg, 3.0 mmol), and a drop of I_2 afforded **1g** as a liquid (430 mg, 49%); ^{1}H NMR (400 MHz, $CDCl_3$) δ 7.17 (d, J = 8.4 Hz, 2 H), 6.92 (d, J = 8.4 Hz, 2 H), 2.30 (s, 2 H), 0.08 (s, 9 H); ^{13}C NMR (100 MHz, $CDCl_3$) δ 141.8, 128.5, 128.3, 127.9, 19.6, -10.1; IR (neat) 1590, 1487, 1407 cm^{-1}; HRMS (EI) calcd for $C_{10}H_{15}Cl^{112}Sn$ 281.9911, found 281.9906.

(6) (4-Fluorobenzyl)trimethylstannane (1h)[2]

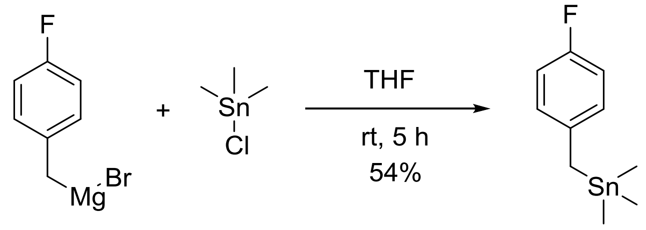

1h

The reaction of 4-fluorobenzyl bromide (564 mg, 3.0 mmol), Mg (157 mg, 6.5 mmol), anhydrous THF (25 mL), trimethyltin chloride (595 mg, 3.0 mmol), and a drop of I_2 afforded **1h** as a liquid (439 mg, 54%); ^{1}H NMR (400 MHz, $CDCl_3$) δ 6.96-6.84 (m, 4 H), 2.28 (s, 2 H), 0.06 (s, 9 H).

(4-(Methoxycarbonyl)benzyl)trimethylstannane (1i)

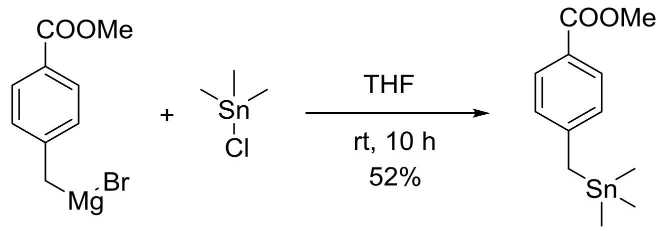

1i

The reaction of 4-(methoxycarbonyl)benzyl bromide (1.268 g, 5.5 mmol), Mg (198 mg, 8.1 mmol), anhydrous THF (15 mL), trimethyltin chloride (589 mg, 3.0 mmol), and a drop of I_2 afforded **1i** as a liquid (482 mg, 52%); ^{1}H NMR (400 MHz, $CDCl_3$) δ 7.86 (d, J = 8.2 Hz, 2 H), 7.00 (d, J = 8.2 Hz, 2 H), 3.85 (s, 3 H), 2.36 (s, 2 H), 0.04 (s, 9 H); ^{13}C NMR (100 MHz, $CDCl_3$) δ 167.2, 149.7, 129.8, 126.3, 124.8, 51.6, 21.1, -10.1; IR (neat) 1718, 1605, 1504, 1432, 1412 cm^{-1}; HRMS (EI) calcd for $C_{12}H_{18}O_2{}^{112}Sn$ 306.0355, found 306.0350.

(7) (4-(Trifluoromethyl)benzyl)trimethylstannane (1j)[3]

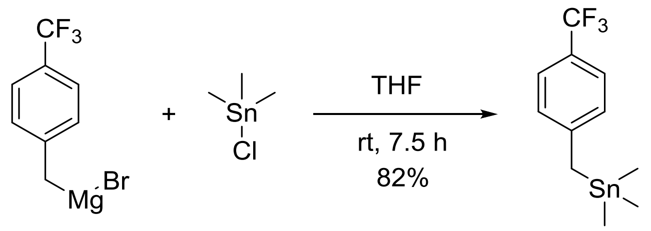

1j

The reaction of 4-(trifluoromethyl)benzyl bromide (717 mg, 3.0 mmol), Mg (143 mg, 6.0 mmol), anhydrous THF (25 mL), trimethyltin chloride (597 mg, 3.0 mmol), and a drop of I_2 afforded **1j** as a liquid (794 mg, 82%); ^{1}H NMR (400 MHz, $CDCl_3$) δ 7.44 (d, J = 8.0 Hz, 2 H), 7.06 (d, J = 8.0 Hz, 2 H), 2.37 (s, 2 H), 0.08 (s, 9 H).

(3-(Trifluoromethyl)benzyl)trimethylstannane (1k)[3]

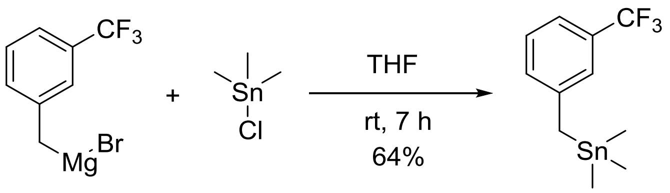

1k

The reaction of 3-(trifluoromethyl)benzyl bromide (610 μl, 4.0 mmol), Mg (187 mg, 7.7 mmol), anhydrous THF (20 mL), trimethyltin chloride (493 mg, 2.5 mmol), and a drop of I_2 afforded **1k** as a liquid (509 mg, 64%); ^{1}H NMR (400 MHz, $CDCl_3$) δ 7.32-7.19 (m, 3 H), 7.13 (d, J = 7.2 Hz, 1 H), 2.36 (s, 2 H), 0.06 (s, 9 H).

(2-(Trifluoromethyl)benzyl)trimethylstannane (1l)

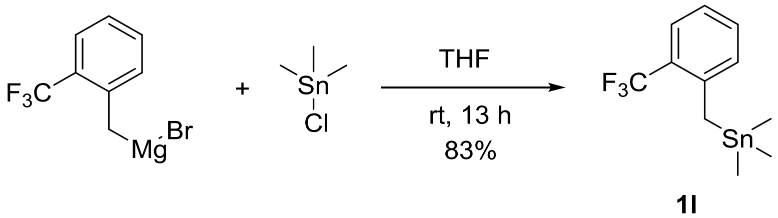

1l

The reaction of 2-(trifluoromethyl)benzyl bromide (600 μl, 3.9 mmol), Mg (173 mg, 7.1 mmol), anhydrous THF (20 mL), trimethyltin chloride (503 mg, 2.5 mmol), and a drop of I_2 afforded **1l** as a liquid (673 mg, 83%); ^{1}H NMR (400 MHz, $CDCl_3$) δ 7.53 (d, J = 7.6 Hz, 1 H), 7.34 (t, J = 7.6 Hz, 1 H), 7.10-7.02 (m, 2 H), 2.51 (d, J = 2.0 Hz, 2 H), 0.05 (s, 9 H); ^{13}C NMR (100 MHz, $CDCl_3$) δ 143.1, 131.5, 129.3, 126.0, 125.9 (q, J = 5.4 Hz), 124.9 (q, J = 271.7 Hz), 122.9, 18.8, -9.5; ^{19}F NMR (376 MHz, $CDCl_3$) δ -61.5 ppm; IR (neat) 1607, 1574, 1488, 1451 cm^{-1}; HRMS (EI) calcd for

$C_{11}H_{15}F_3{}^{112}Sn$ 316.0174, found 316.0178.

(8) (1-Naphthyl)trimethylstannane (1m)

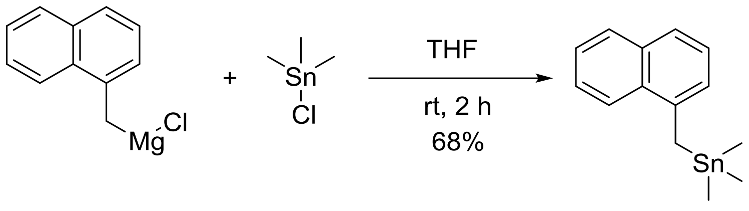

1m

The reaction of 1-chloromethyl naphthalene (0.7 mL, 4.7 mmol), Mg (166 mg, 6.8 mmol), anhydrous THF (25 mL), trimethyltin chloride (607 mg, 3.0 mmol), and a drop of I_2 afforded **1m** as a liquid (632 mg, 68%); ^{1}H NMR (400 MHz, $CDCl_3$) δ 8.07-7.93 (m, 2 H), 7.70 (d, J = 8.0 Hz, 1 H), 7.65-7.55 (m, 2 H), 7.48 (t, J = 7.6 Hz, 1 H), 7.31 (d, J = 7.2 Hz, 1 H), 2.93 (s, 2 H), 0.16 (s, 9 H); ^{13}C NMR (100 MHz, $CDCl_3$) δ 139.7, 133.9, 130.9, 128.7, 125.7, 125.3, 125.0, 124.1, 123.9, 123.6, 17.8, -9.2; IR (neat) 1587, 1574, 1506, 1459, 1441 cm^{-1}; HRMS (EI) calcd for $C_{14}H_{18}{}^{112}Sn$ 298.0457, found 298.0454.

(9) (2-Naphthyl)trimethylstannane (1n)

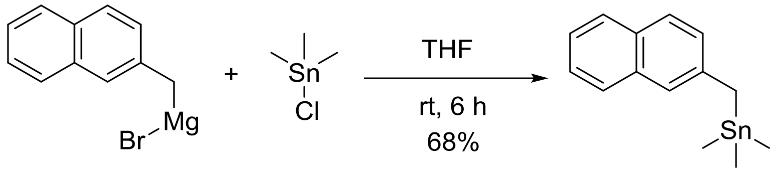

1n

The reaction of 2-bromomethyl naphthalene (737 mg, 3.3 mmol), Mg (172 mg, 7.1 mmol), anhydrous THF (25 mL), trimethyltin chloride (532 mg, 2.7 mmol), and a drop of I_2 afforded **1n** as a liquid (554 mg, 68%); ^{1}H NMR (400 MHz, $CDCl_3$) δ 7.77 (d, J = 8.4 Hz, 1 H), 7.71 (d, J = 8.4 Hz, 2 H), 7.47-7.39 (m, 2 H), 7.38-7.31 (m, 1 H), 7.16 (d, J = 8.4 Hz, 1 H), 2.51 (s, 2 H), 0.09 (s, 9 H); ^{13}C NMR (100 MHz, $CDCl_3$) δ 140.9, 134.0, 130.6, 127.8, 127.6, 127.0, 126.8, 125.8, 124.0, 123.3, 20.6, -9.9; IR (neat) 1627, 1598, 1505, 1468, 1436 cm^{-1}; HRMS (EI) calcd for $C_{14}H_{18}{}^{112}Sn$ 298.0457, found 298.0463.

(10) (2-(Phenyl)ethyl)trimethylstannane (1o)[4]

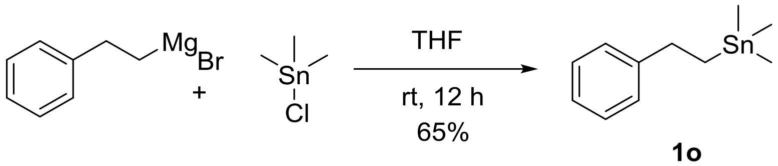

The reaction of (2-bromoethyl)benzene (550 μL, 4.0 mmol), Mg (186 mg, 7.7 mmol), anhydrous THF (24 mL), trimethyltin chloride (603 mg, 3.0 mmol), and a drop of I_2 afforded **1o** as a liquid (532 mg, 65%); ^{1}H NMR (400 MHz, $CDCl_3$) δ 7.33-7.26 (m, 2 H), 7.24-7.14 (m, 3 H), 2.85 (t, J = 8.4 Hz, 2 H), 1.18 (t, J = 8.4 Hz, 2 H), 0.02 (s, 9 H).

Typical Procedure II for the photoreaction.

Synthesis of 4-methoxybenzoic acid (3a)[5]

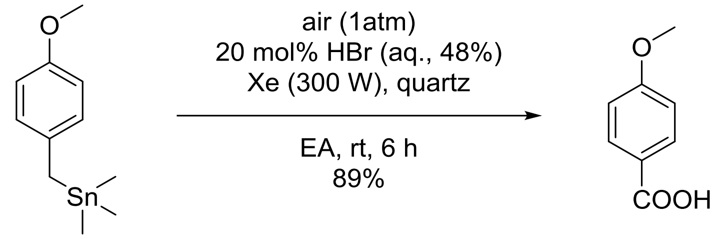

1a **3a**

1a (57 mg, 0.20 mmol), EA (10 mL), and HBr (aq., 48%) (4.5 μL, 0.04 mmol) were added to a quartz reaction flask which was equipped with a magnetic stirrer and a condenser. The mixture was irradiated by a Xe lamp (300 W) at rt in the open air. The photoreaction was completed after 6 hours as monitored by TLC (eluent: petroleum ether : ethyl acetate = 10:1). The solvent was removed and the residue was purified by flash chromatography on silica gel (eluent: petroleum ether : ethyl acetate = 3:1) to afford **3a** as a solid (27 mg, 89%); ^{1}H NMR (400 MHz, $CDCl_3$) δ 8.07 (d, J = 8.4 Hz, 2 H), 6.95 (d, J = 8.4 Hz, 2 H), 3.88 (s, 3 H).

The following compounds were prepared according to typical procedure II.

(1) 3-Methoxybenzoic acid (3b)[5]

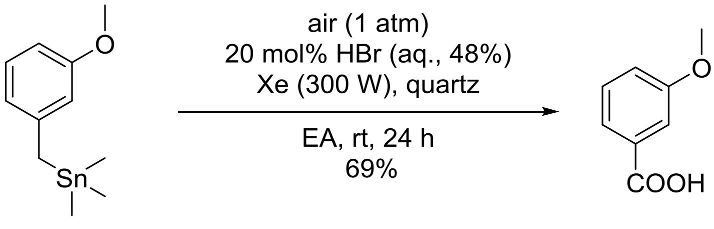

1b **3b**

The reaction of **1b** (57 mg, 0.20 mmol) and HBr (aq., 48%) (4.5 µL, 0.04 mmol) in EA (10 mL) afforded **3b** as a solid (21 mg, 69%); ^{1}H NMR (400 MHz, $CDCl_3$) δ 7.73 (d, J = 8.0 Hz, 1 H), 7.63 (s, 1 H), 7.39 (t, J = 8.0 Hz, 1 H), 7.16 (dd, J = 8.4, 2.8 Hz 1 H), 3.87 (s, 3 H).

(2) 2-Methoxybenzoic acid (3c)[5]

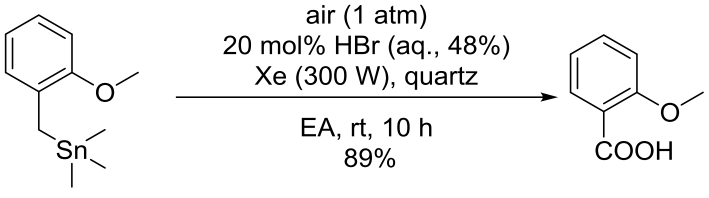

1c **3c**

The reaction of **1c** (57 mg, 0.20 mmol) and HBr (aq., 48%) (4.5 µL, 0.04 mmol) in EA (10 mL) afforded **3c** as a solid (27 mg, 89%); ^{1}H NMR (400 MHz, $CDCl_3$) δ 8.20 (dd, J = 8.0, 2.0 Hz, 1 H), 7.63-7.55 (m, 1 H), 7.16 (td, J = 7.6, 0.8 Hz, 1 H), 7.07 (dd, J = 8.4, 0.8 Hz, 1 H), 4.09 (s, 3 H).

(3) 4-*Tert*-butylbenzoic acid (3d)[5]

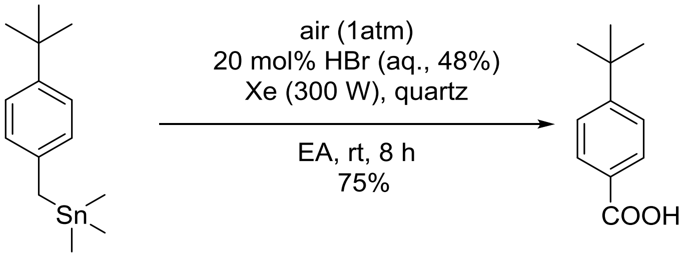

1d **3d**

The reaction of **1d** (63 mg, 0.20 mmol) and HBr (aq., 48%) (4.5 µL, 0.04 mmol) in EA (10 mL) afforded **3d** as a solid (27 mg, 75%); ^{1}H NMR (400 MHz, $CDCl_3$) δ 8.06 (d, J = 8.4 Hz, 2 H), 7.49 (d, J = 8.4 Hz, 2 H), 1.35 (s, 9 H).

(4) 4-Phenylbenzoic acid (3e)[5]

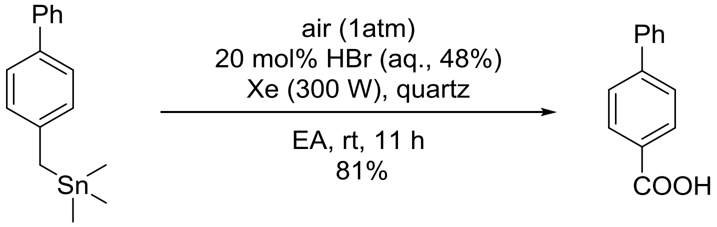

1e **3e**

The reaction of **1e** (66 mg, 0.20 mmol) and HBr (aq., 48%) (4.5 µL, 0.04 mmol) in EA (10 mL) afforded **3e** as a solid (32 mg, 81%); ^{1}H NMR (400 MHz, $CDCl_3$) δ

8.18-8.09 (m, 2 H), 7.71-7.60 (m, 4 H), 7.52-7.32 (m, 3 H).

(5) Benzoic acid (3f)[5]

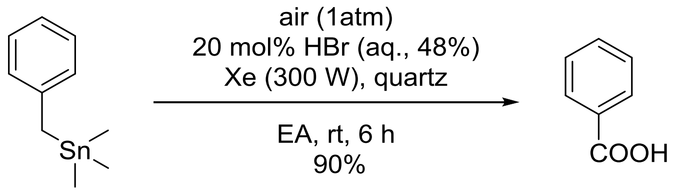

1f **3f**

The reaction of **1f** (51 mg, 0.20 mmol) and HBr (aq., 48%) (4.5 μL, 0.04 mmol) in EA (10 mL) afforded **3f** as a solid (22 mg, 90%); ^{1}H NMR (400 MHz, DMSO-d_6) δ 7.97 (dd, J = 8.0, 1.2 Hz, 2 H), 7.67-7.59 (m, 1 H), 7.55-7.47 (m, 2 H).

(6) 4-Chlorobenzoic acid (3g)[5]

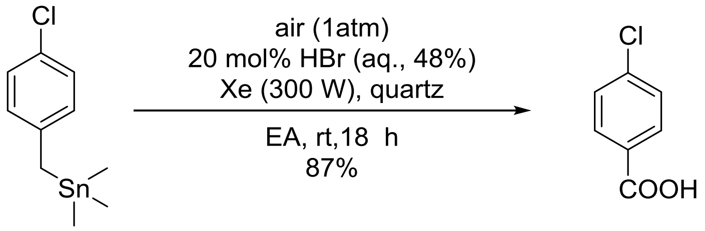

1g **3g**

The reaction of **1g** (58 mg, 0.20 mmol) and HBr (aq., 48%) (4.5 μL, 0.04 mmol) in EA (10 mL) afforded **3g** as a solid (27 mg, 87%); ^{1}H NMR (400 MHz, $CDCl_3$) δ 8.04 (d, J = 8.4 Hz, 2 H), 7.46 (d, J = 8.4 Hz, 2 H).

(7) 4-Fluorobenzoic acid (3h)[5]

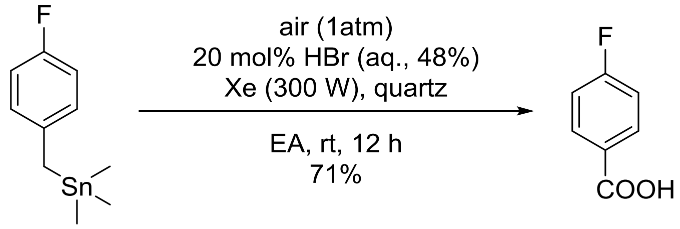

1h **3h**

The reaction of **1h** (55 mg, 0.20 mmol) and HBr (aq., 48%) (4.5 μL, 0.04 mmol) in EA (10 mL) afforded **3h** as a solid (20 mg, 71%); ^{1}H NMR (400 MHz, $CDCl_3$) δ 8.18-8.10 (m, 2 H), 7.15-7.11 (m, 2 H).

(8) 4-(Methoxycarbonyl)benzoic acid (3i)[5]

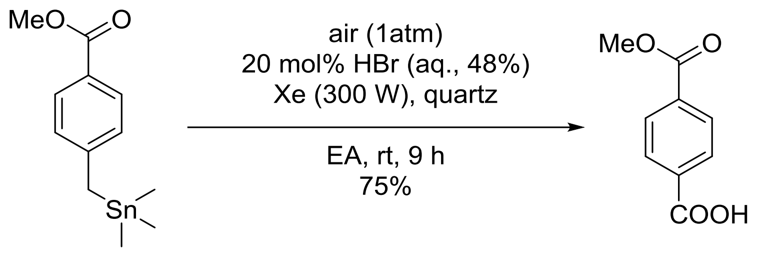

1i **3i**

The reaction of **1i** (63 mg, 0.20 mmol) and HBr (aq., 48%) (4.5 μL, 0.04 mmol) in EA (10 mL) afforded **3i** as a solid (27 mg, 75%); ^{1}H NMR (400 MHz, DMSO-d_6) δ 8.07 (s, 4 H), 3.89 (s, 3 H).

(9) 4-(Trifluoromethyl)benzoic acid (3j)[5]

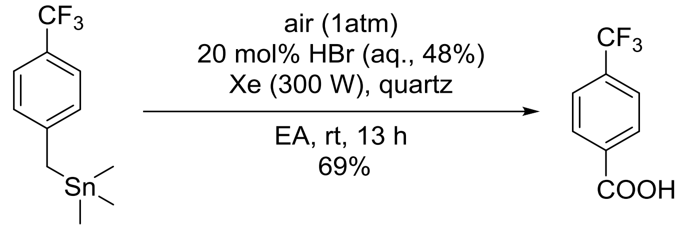

1j **3j**

The reaction of **1j** (67 mg, 0.21 mmol) and HBr (aq., 48%) (4.5 μL, 0.04 mmol) in EA (10 mL) afforded **3j** as a solid (27 mg, 69%); ^{1}H NMR (400 MHz, $CDCl_3$) δ 8.00 (d, J = 8.0 Hz, 2 H), 7.54 (d, J = 8.2 Hz, 2 H).

(10) 3-(Trifluoromethyl)benzoic acid (3k)[5]

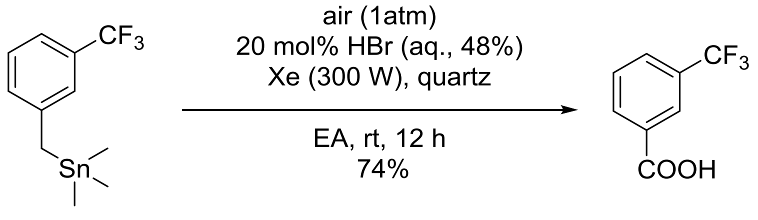

1k **3k**

The reaction of **1k** (65 mg, 0.20 mmol) and HBr (aq., 48%) (4.5 μL, 0.04 mmol) in EA (10 mL) afforded **3k** as a solid (28 mg, 74%); ^{1}HNMR (400 MHz, DMSO-d_6): δ 8.25-8.14 (m, 2 H), 7.98 (d, J = 7.2 Hz, 1 H), 7.78-7.71 (m, 1 H).

(11) 2-(Trifluoromethyl)benzoic acid (3l)[5]

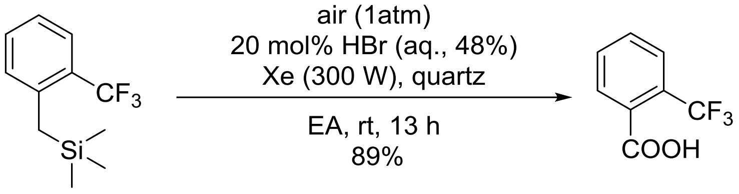

1l **3l**

The reaction of **1l** (65 mg, 0.20 mmol) and HBr (aq., 48%) (4.5 μL, 0.04 mmol) in EA (10 mL) afforded **3l** as a solid (34 mg, 89%); ^{1}H NMR (400 MHz, $CDCl_3$) δ 8.02-7.96 (m, 1 H), 7.84-7.78 (m, 1 H), 7.70-7.63 (m, 2 H).

(12) 1-Naphthoic acid (3m)[6]

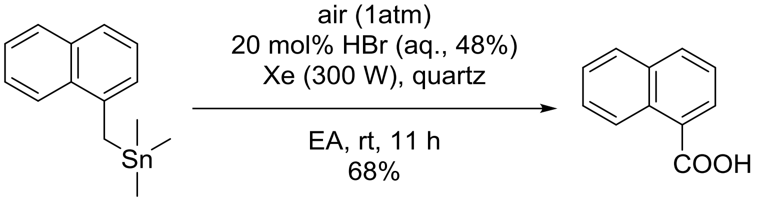

1m **3m**

The reaction of **1m** (65 mg, 0.21 mmol) and HBr (aq., 48%) (4.5 μL, 0.04 mmol) in EA (10 mL) afforded **3m** as a solid (25 mg, 68%); ^{1}H NMR (400 MHz, DMSO-d_6) δ 13.20 (brs, 1 H), 8.89 (d, J = 8.8 Hz, 1 H), 8.20-8.14 (m, 2 H), 8.03 (d, J = 8.0 Hz, 1 H), 7.70-7.57 (m, 3 H).

(13) 2-Naphthoic acid (3n)[7]

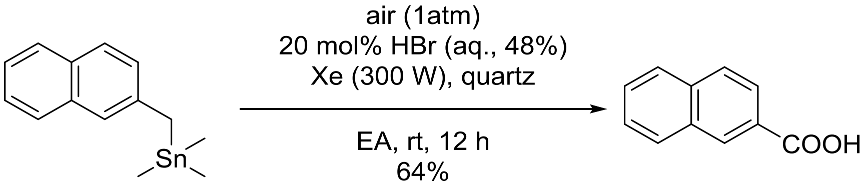

1n **3n**

The reaction of **1n** (61 mg, 0.20 mmol) and HBr (aq., 48%) (4.5 μL, 0.04 mmol) in EA (10 mL) afforded **3n** as a solid (22 mg, 64%); ^{1}H NMR (400 MHz, DMSO-d_6) δ 13.15 (brs, 1 H), 8.63 (s, 1 H), 8.13 (d, J = 7.6 Hz, 1 H), 8.06-7.96 (m, 3 H), 7.71-7.58 (m, 2 H).

(14) benzoic acid (3f)

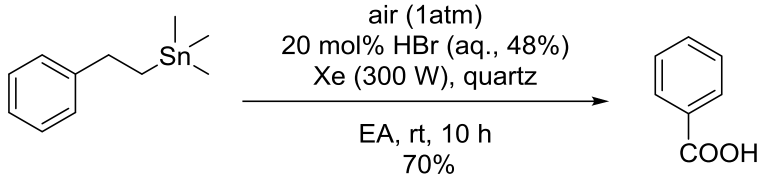

1o **3f**

The reaction of **6** (54 mg, 0.20 mmol) and HBr (aq., 48%) (4.5 µL, 0.04 mmol) in EA (10 mL) afforded **3f** as a solid (17 mg, 70%).

(15) Acetophenone (3p)[8]

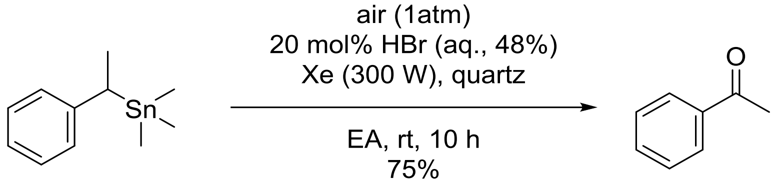

1p **3p**

The reaction of **1p** (54 mg, 0.20 mmol) and HBr (aq., 48%) (4.5 µL, 0.04 mmol) in EA (10 mL) afforded **3p** as a liquid (18 mg, 75%); ^{1}H NMR (400 MHz, $CDCl_3$) δ 7.99-7.93 (m, 2 H), 7.59-7.51 (m, 1 H), 7.50-7.45 (m, 2 H), 2.61 (s, 3 H).

(15)Acetophenone (3q)[8]

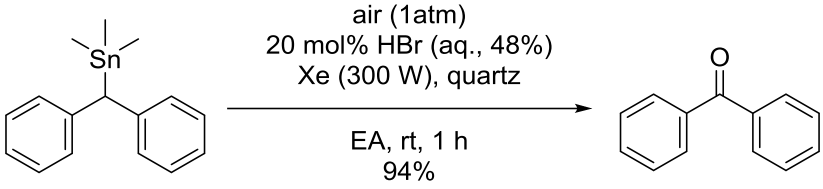

1q **3q**

The reaction of **1q** (66 mg, 0.20 mmol) and HBr (aq., 48%) (4.5 µL, 0.04 mmol) in EA (10 mL) afforded **3q** as a solid (34 mg, 94%); ^{1}H NMR (400 MHz, $CDCl_3$) δ 7.81 (d, J = 7.2 Hz, 4 H), 7.63-7.45 (m, 6 H).

Photo reaction of 2a under Condition A

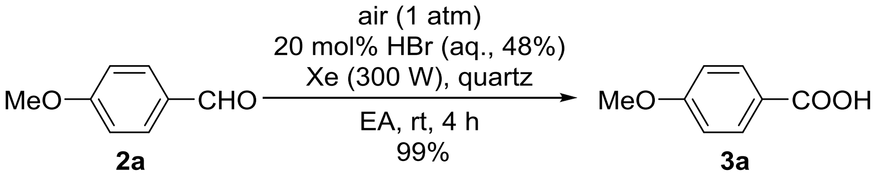

2a **3a**

2a (27 mg, 0.20 mmol), EA (10 mL), and HBr (aq., 48%) (4.5 µL, 0.04 mmol) were added to a quartz reaction flask which was equipped with a magnetic stirrer and a

condenser. The mixture was irradiated by a Xe lamp (300 W) at rt in the open air. The photoreaction was completed after 4 hours as monitored by TLC (eluent: petroleum ether : ethyl acetate = 10:1). The solvent was removed and the residue was purified by flash chromatography on silica gel (eluent: petroleum ether : ethyl acetate = 3:1) to afford **3a** as a solid (30 mg, 99%).

Br_2-catalyzed photo reaction of 1a

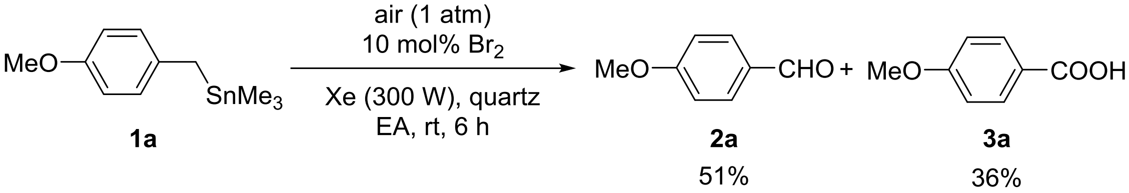

1a (57 mg, 0.20 mmol), EA (10 mL), and Br_2 (1.0 μL, 0.02 mmol) were added to a quartz reaction flask which was equipped with a magnetic stirrer and a condenser. The mixture was irradiated by a Xe lamp (300 W) at rt in the open air. The photoreaction was completed after 6 hours as monitored by TLC (eluent: petroleum ether : ethyl acetate = 10:1). The solvent was removed and the residue was purified by flash chromatography on silica gel (eluent: petroleum ether : ethyl acetate = 3:1) to afford **2a** (14 mg, 51%) and **3a** (11 mg, 36%).

NMR Spectra

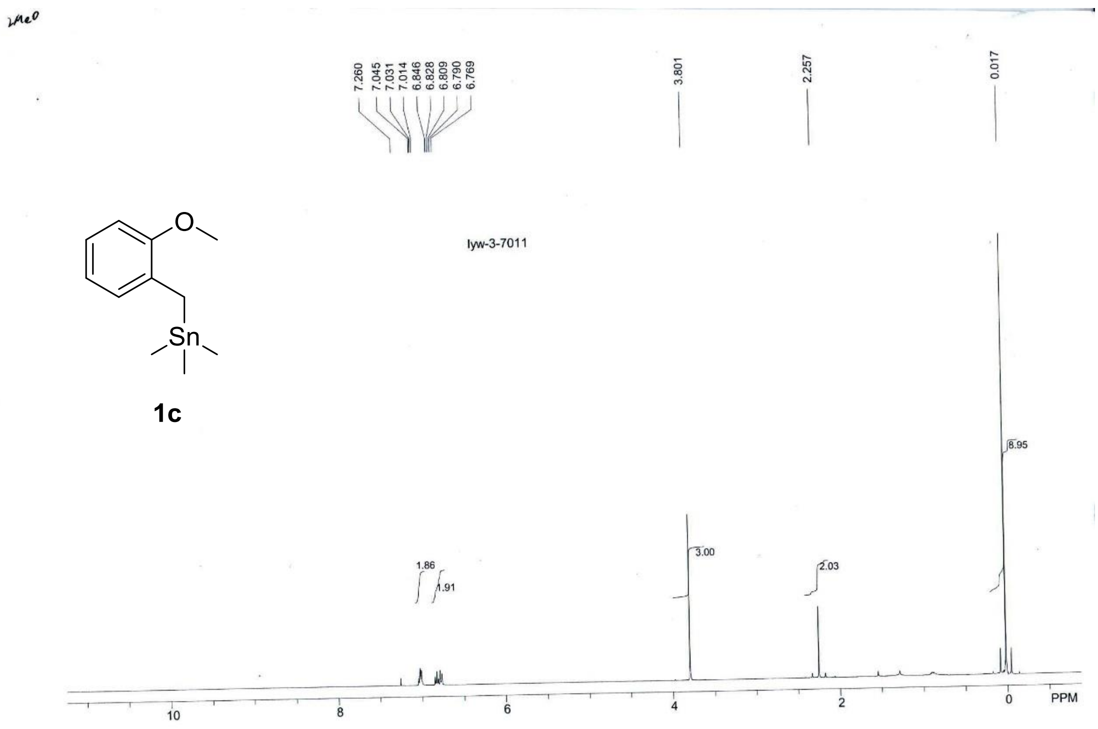

1c

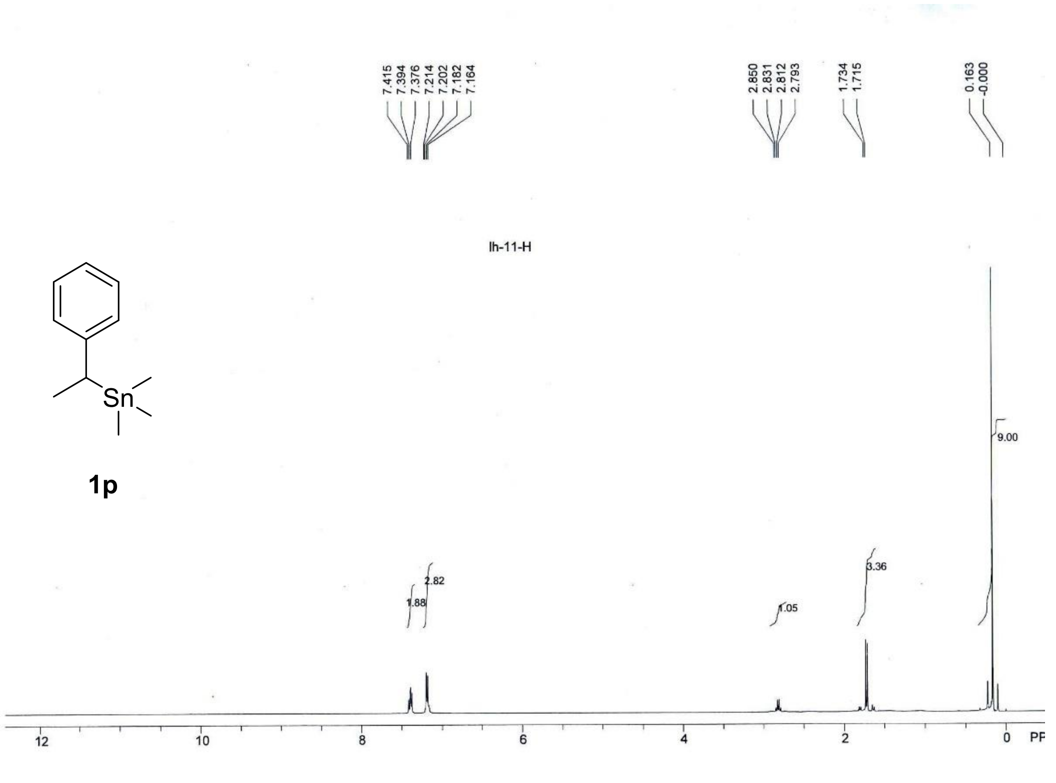

1p

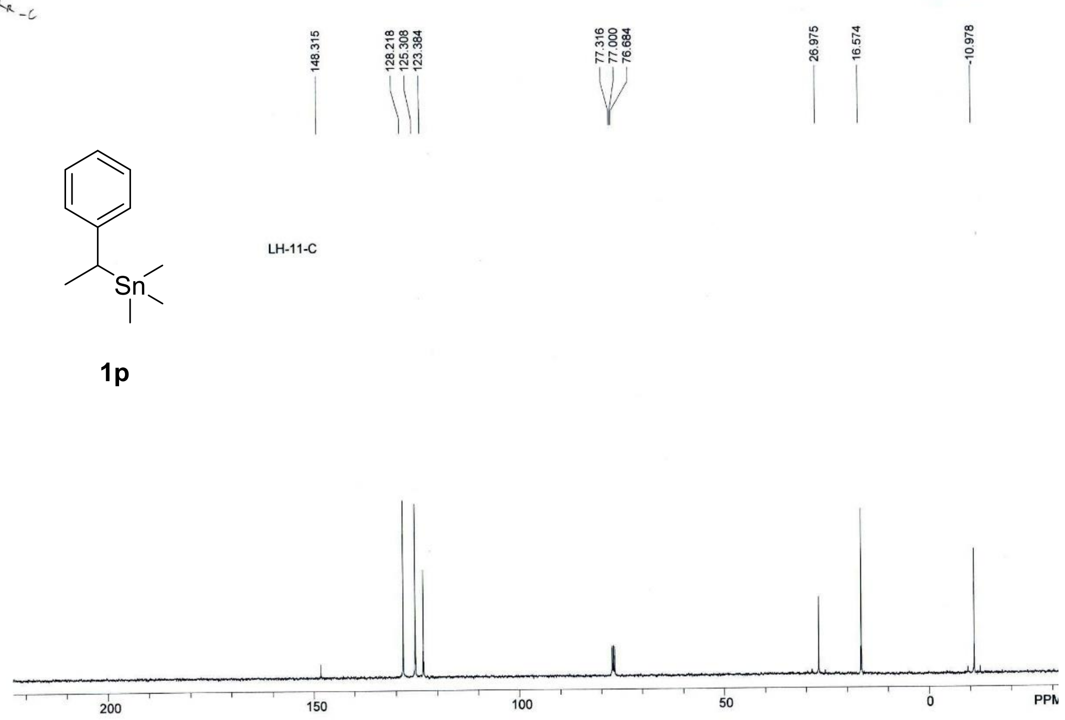

1p
148.315
128.218
125.308
123.384
77.316
77.000
76.684
26.975
16.574
-10.978
LH-11-C
200
150
100
50
0
PPM

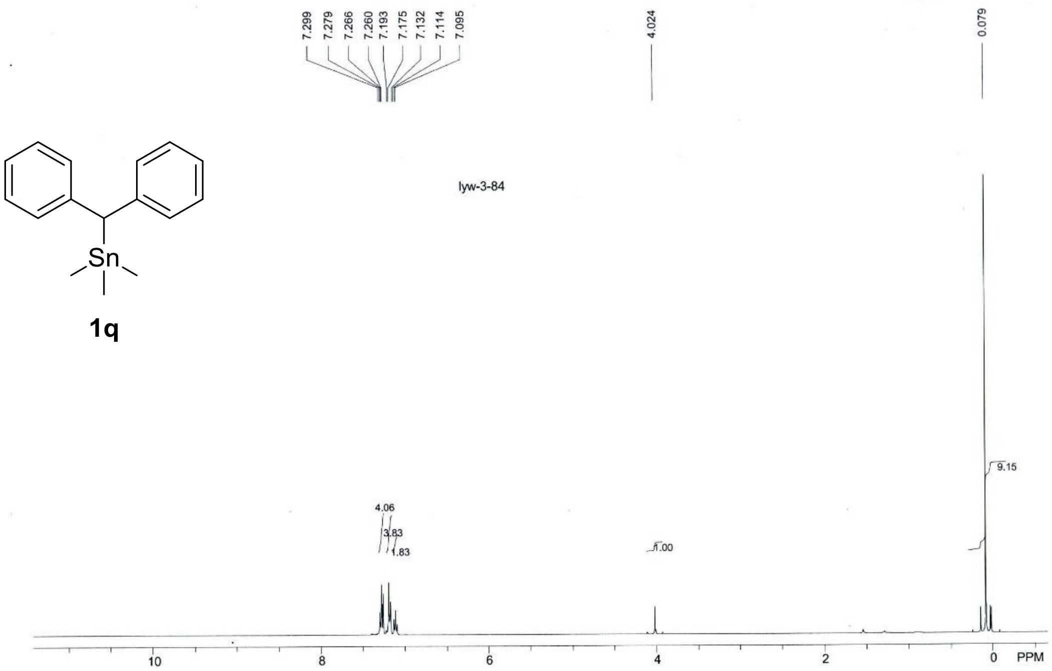

1q
7.299
7.279
7.266
7.260
7.193
7.175
7.132
7.114
7.095
4.024
0.079
lyw-3-84
9.15
4.06
3.83
1.83
1.00
10
8
6
4
2
0
PPM

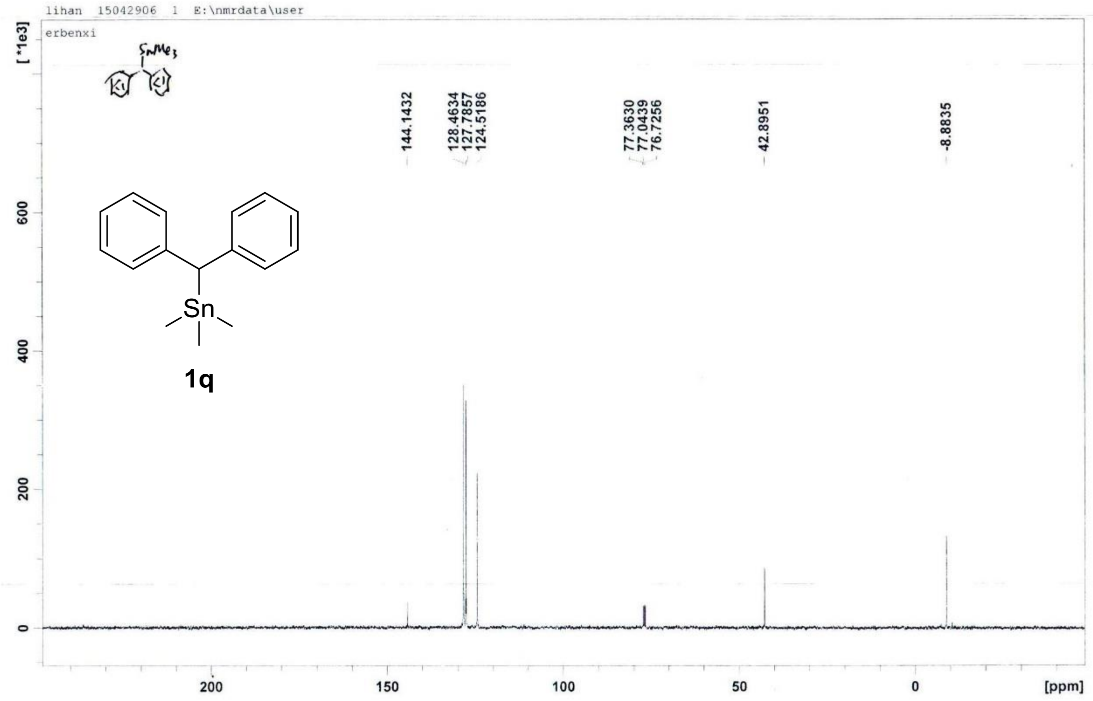
lihan 15042906 1 E:\nmrdata\user
erbenxi
[*1e3]
144.1432
128.4634
127.7857
124.5186
77.3630
77.0439
76.7256
42.8951
-8.8835
600
400
200
0
Sn
1q
200
150
100
50
0
[ppm]

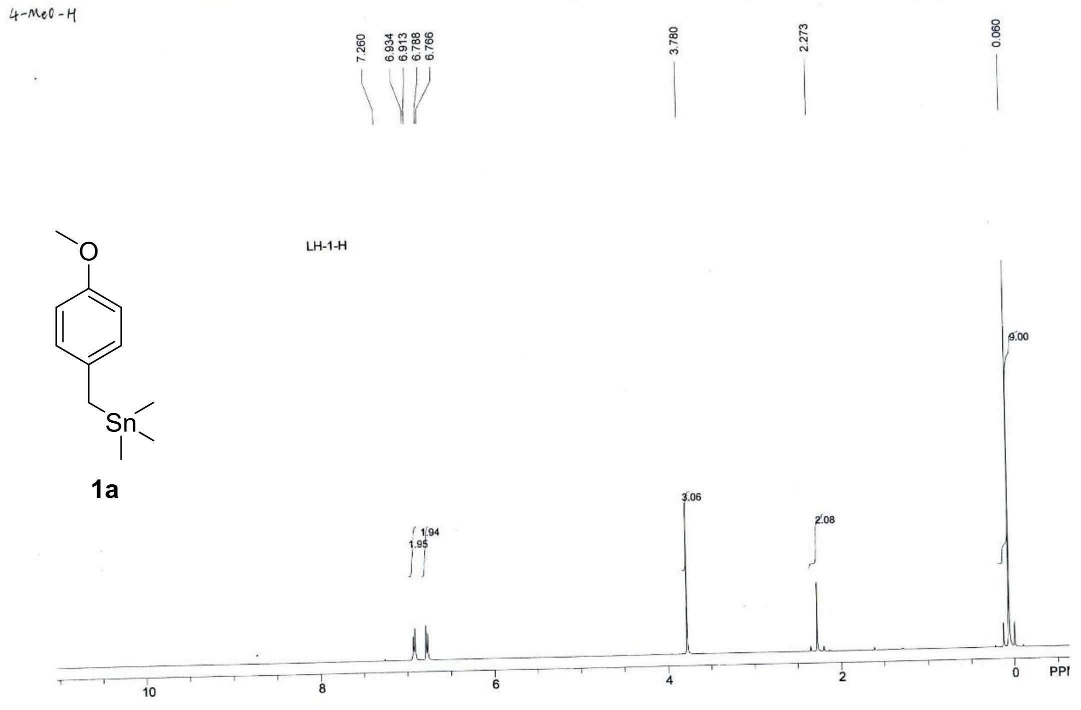
4-MeO-H
7.260
6.934
6.913
6.788
6.766
3.780
2.273
0.060
LH-1-H
O
Sn
1a
9.00
3.06
2.08
1.94
1.95
10
8
6
4
2
0

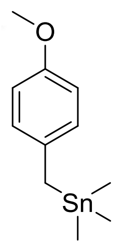

1a

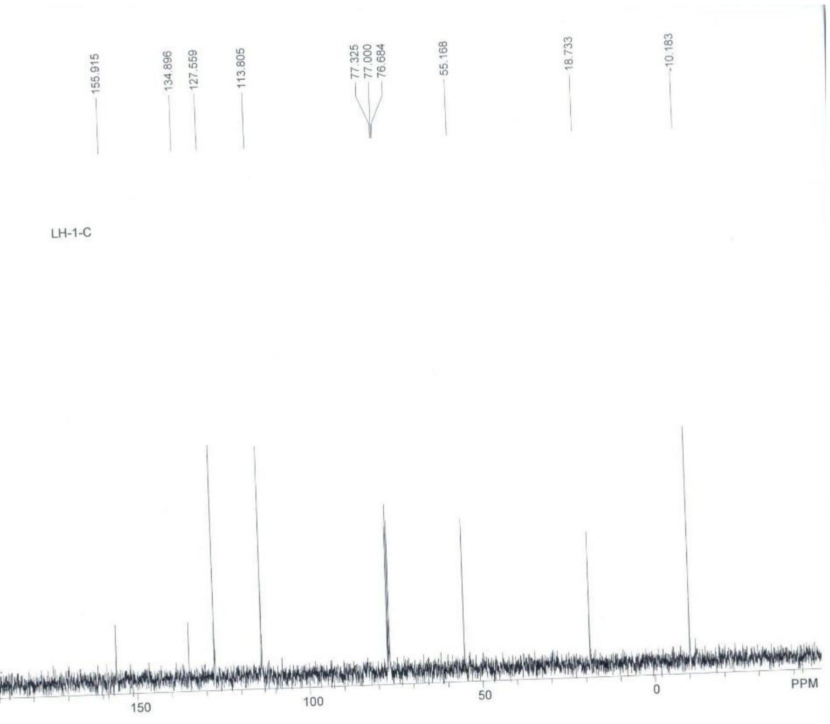
4-MeO-C
155.915
134.896
127.559
113.805
77.325
77.000
76.684
55.168
18.733
-10.183
LH-1-C
200
150
100
50
0
PPM

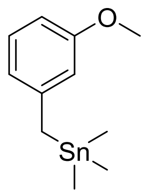

1b

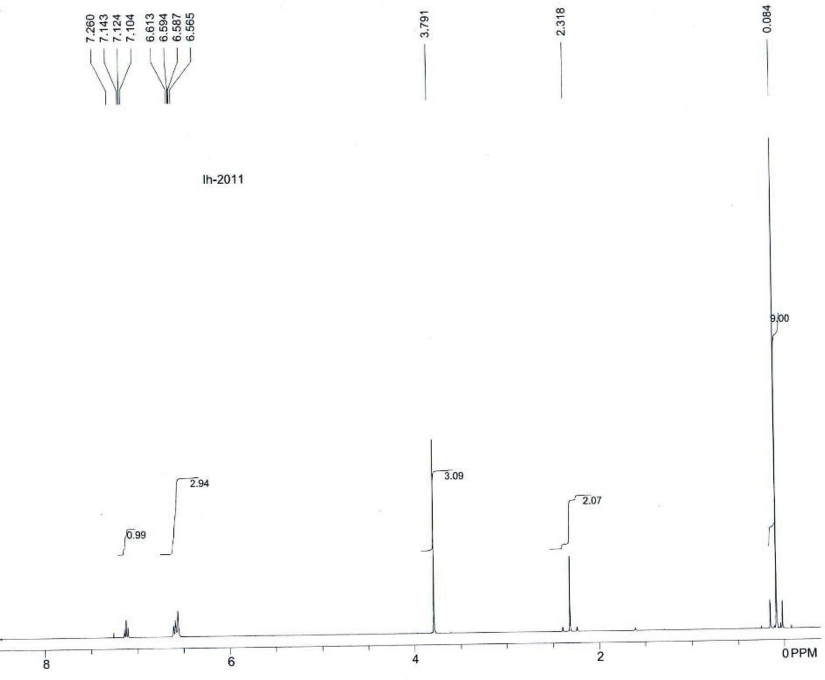
7.260
7.143
7.124
7.104
6.613
6.594
6.587
6.565
3.791
2.318
0.084
lh-2011
9.00
3.09
2.07
2.94
0.99
10
8
6
4
2
0 PPM

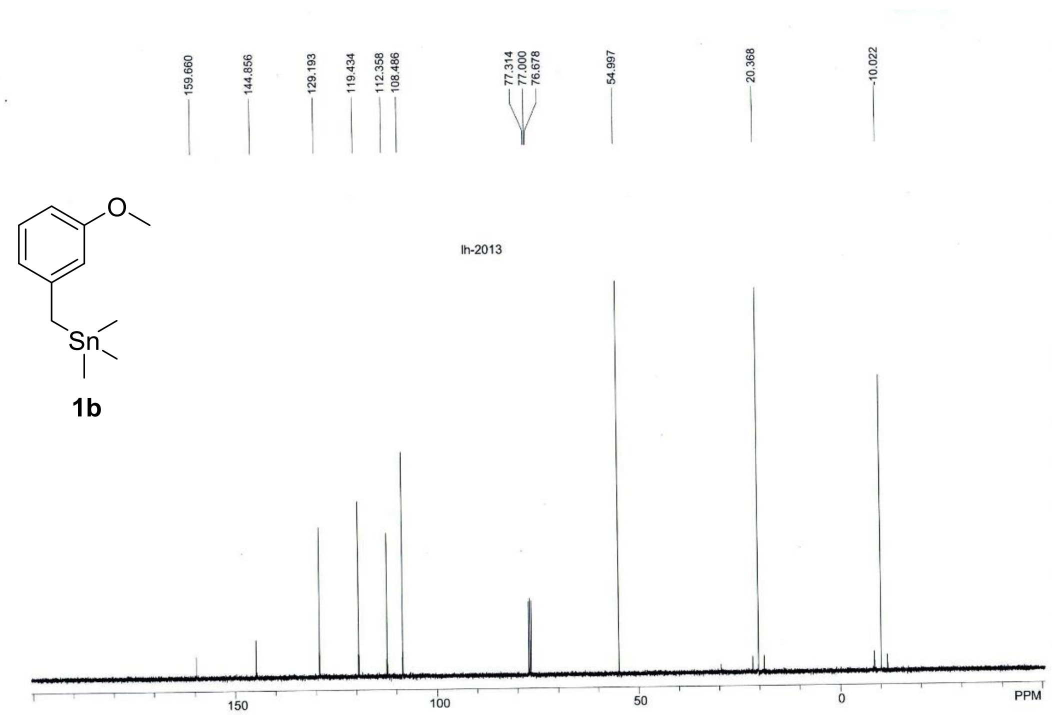
159.660
144.856
129.193
119.434
112.358
108.486
77.314
77.000
76.678
54.997
20.368
-10.022
lh-2013
150
100
50
0
PPM

1b

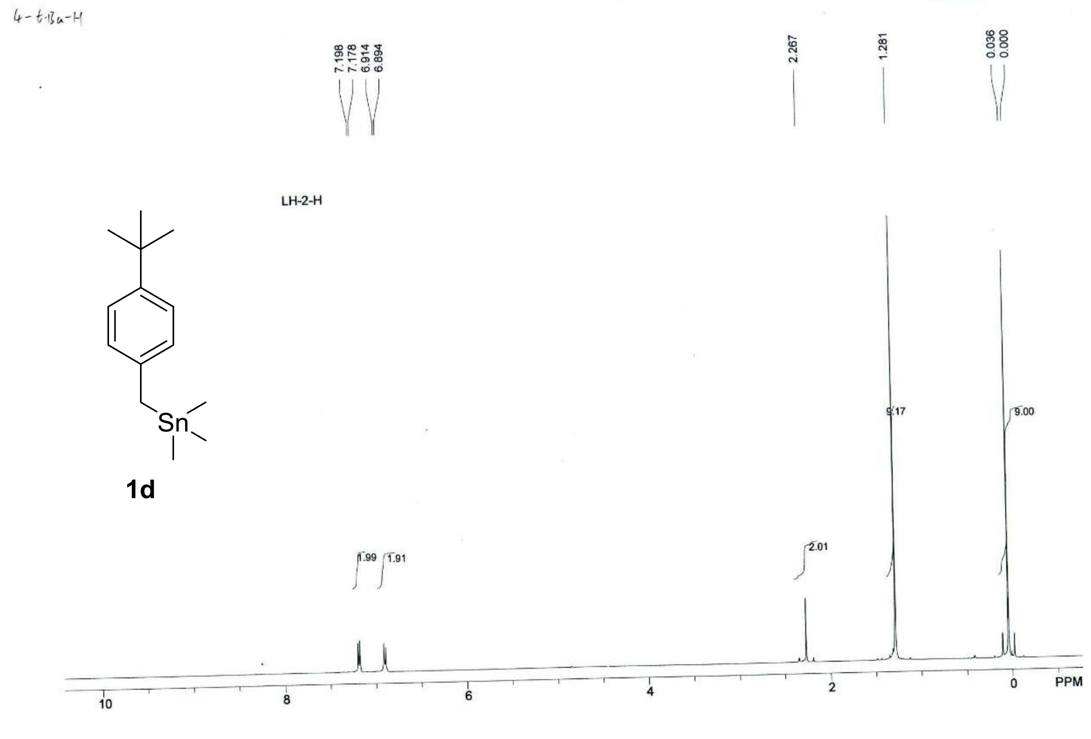
4-t-Bu-H
7.198
7.178
6.914
6.894
2.267
1.281
0.036
0.000
LH-2-H
1.99
1.91
2.01
9.17
9.00
10
8
6
4
2
0
PPM

1d

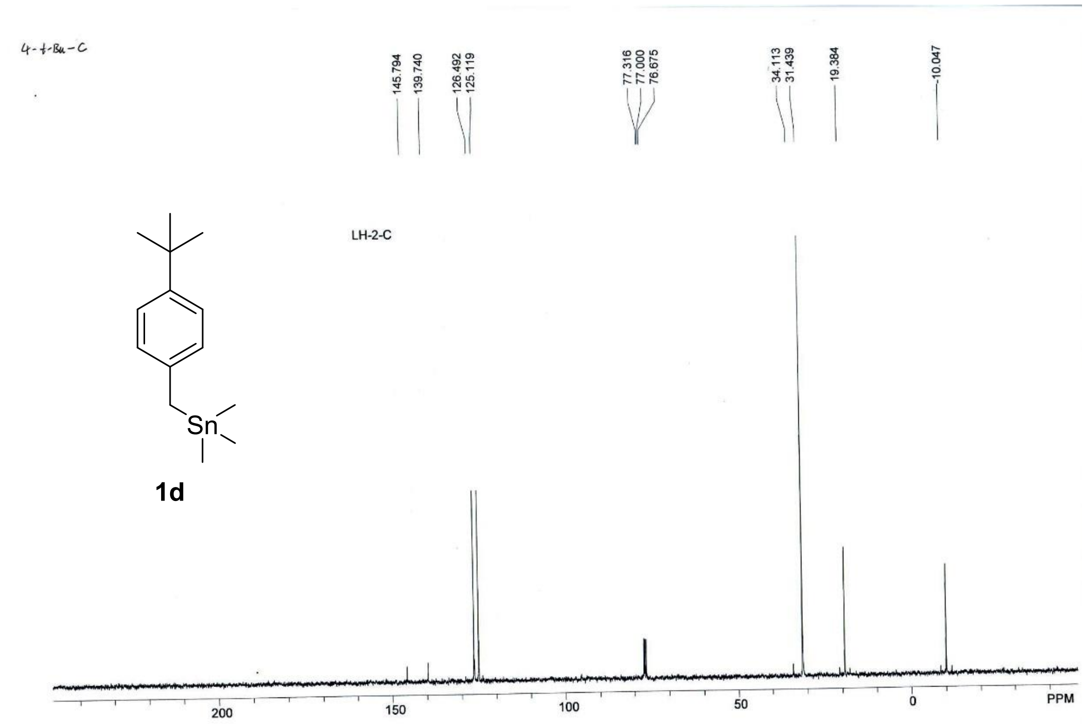
4-t-Bu-C
145.794
139.740
126.492
125.119
77.316
77.000
76.675
34.113
31.439
19.384
-10.047
LH-2-C
Sn
1d
200
150
100
50
0
PPM

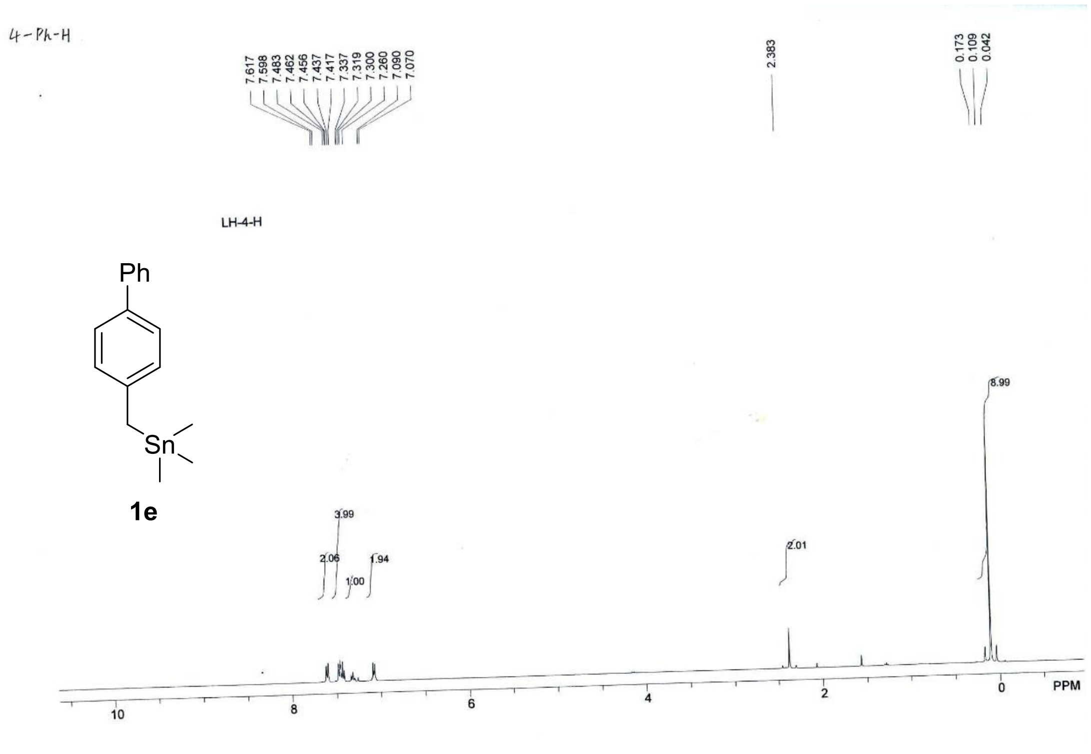
4-Ph-H
7.617
7.598
7.483
7.462
7.456
7.437
7.417
7.337
7.319
7.300
7.260
7.090
7.070
2.383
0.173
0.109
0.042
LH-4-H
Ph
Sn
1e
3.99
2.06
1.00
1.94
2.01
8.99
10
8
6
4
2
0
PPM

4-Ph-C

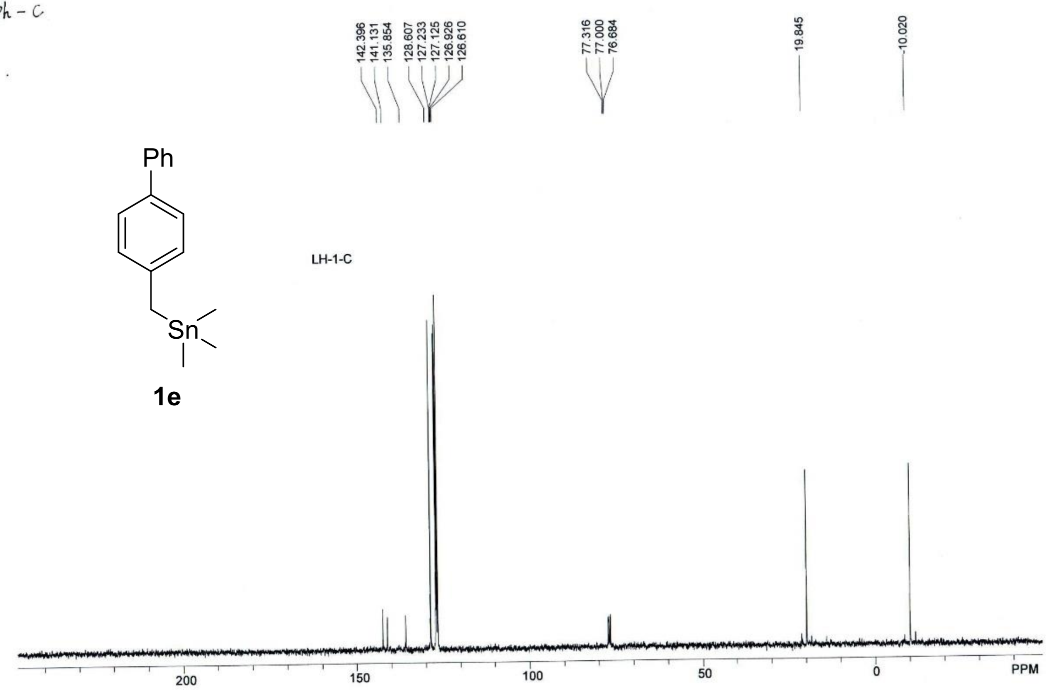

1e

4-H-H

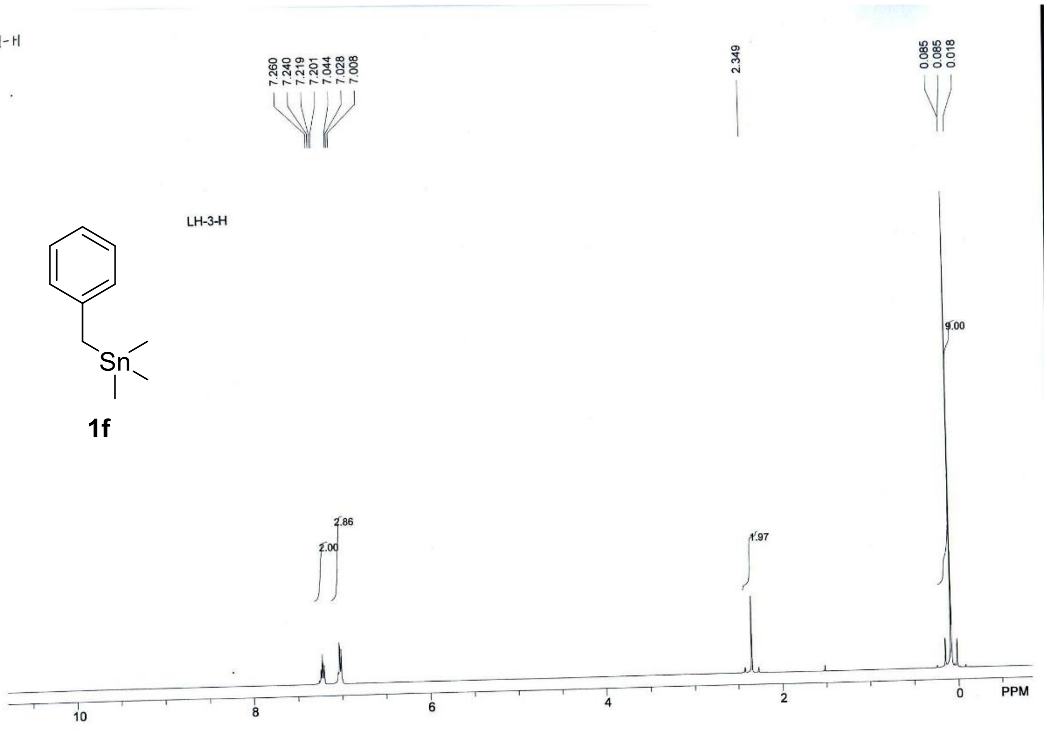

1f

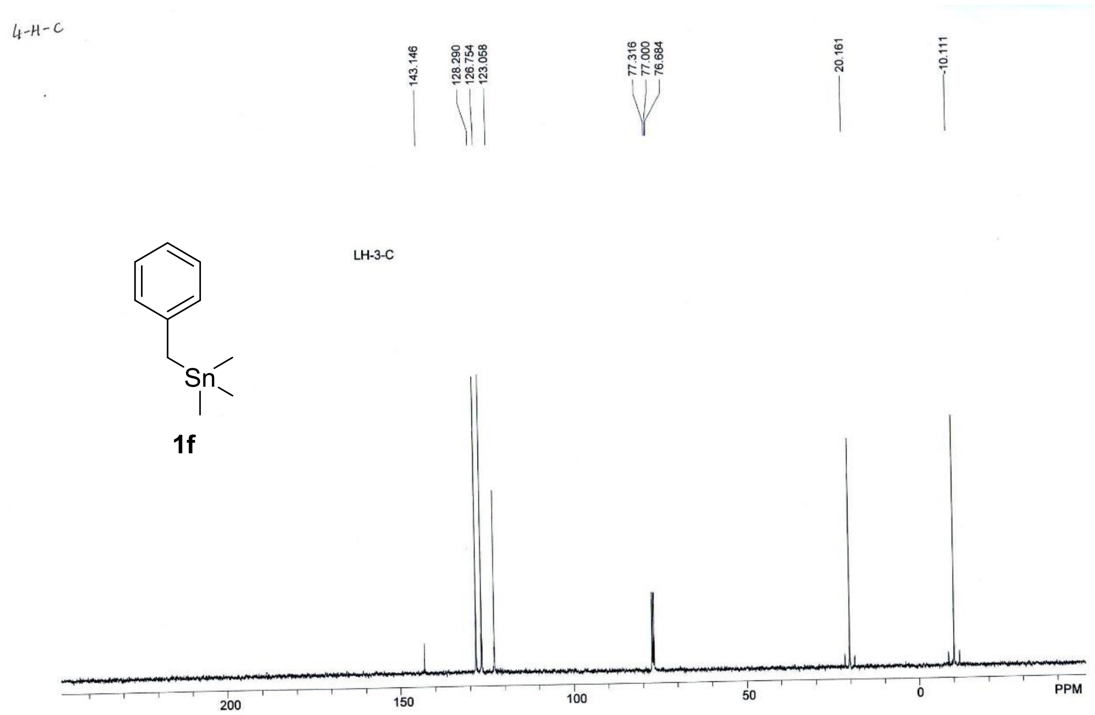
4-H-C
143.146
128.290
126.754
123.058
77.316
77.000
76.684
20.161
-10.111
LH-3-C
200
150
100
50
0
PPM
Sn

1f

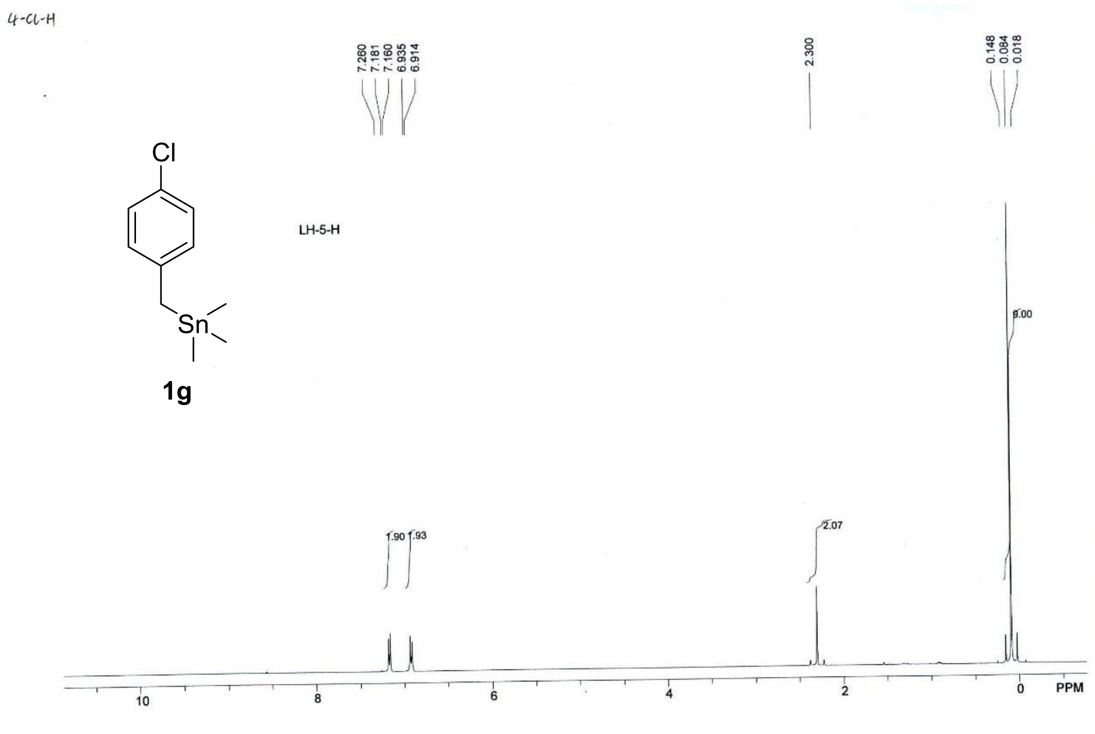
4-Cl-H
7.260
7.181
7.160
6.935
6.914
2.300
0.148
0.084
0.018
LH-5-H
9.00
1.90
1.93
2.07
10
8
6
4
2
0
PPM
Cl
Sn

1g

4-Cl-C

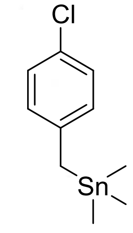

1g

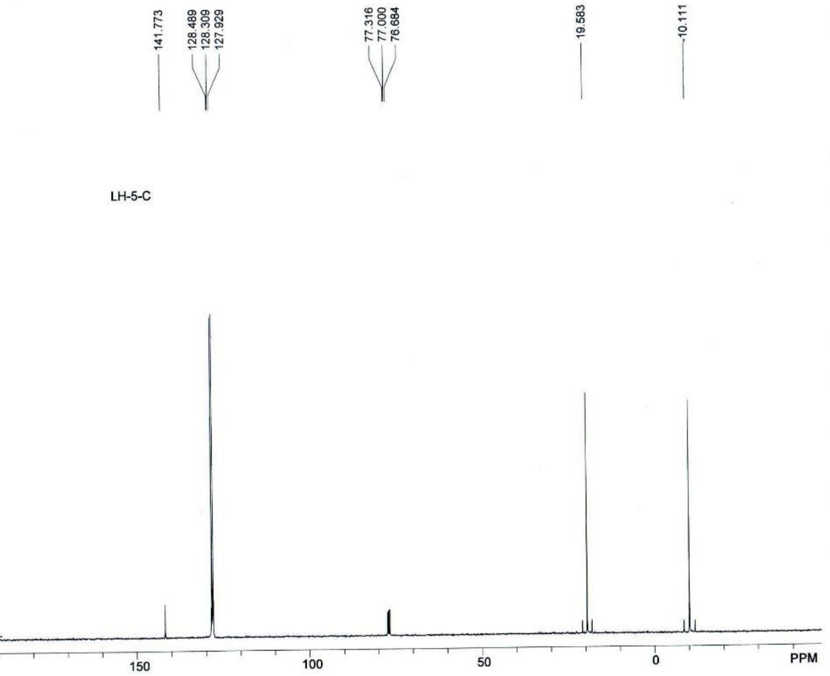

141.773
128.489
128.309
127.929
77.316
77.000
76.684
19.583
-10.111
LH-5-C
200
150
100
50
0
PPM

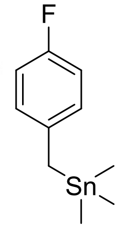

1h

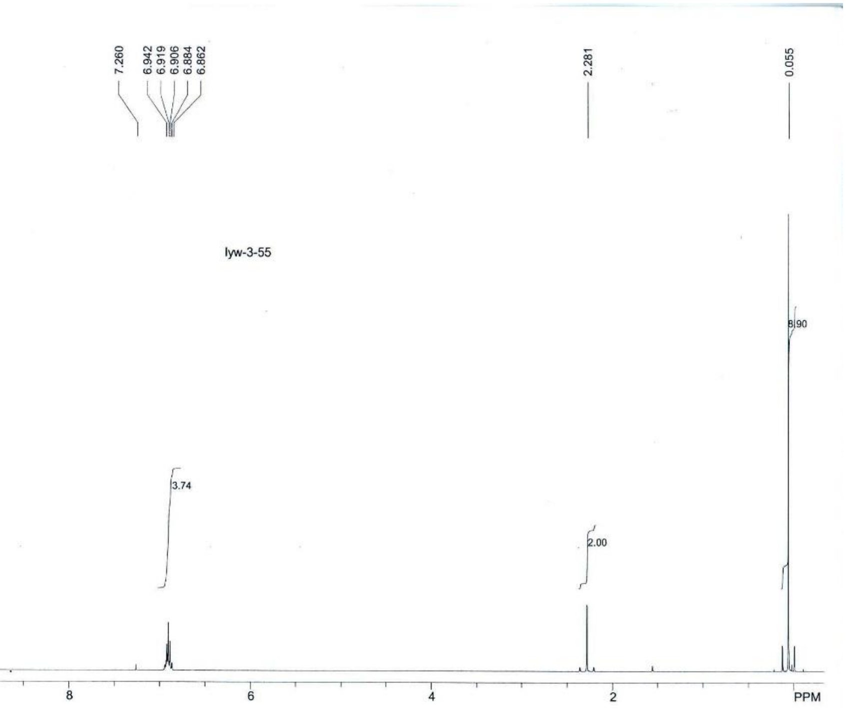

7.260
6.942
6.919
6.906
6.884
6.862
2.281
0.055
lyw-3-55
3.74
2.00
8.90
10
8
6
4
2
PPM

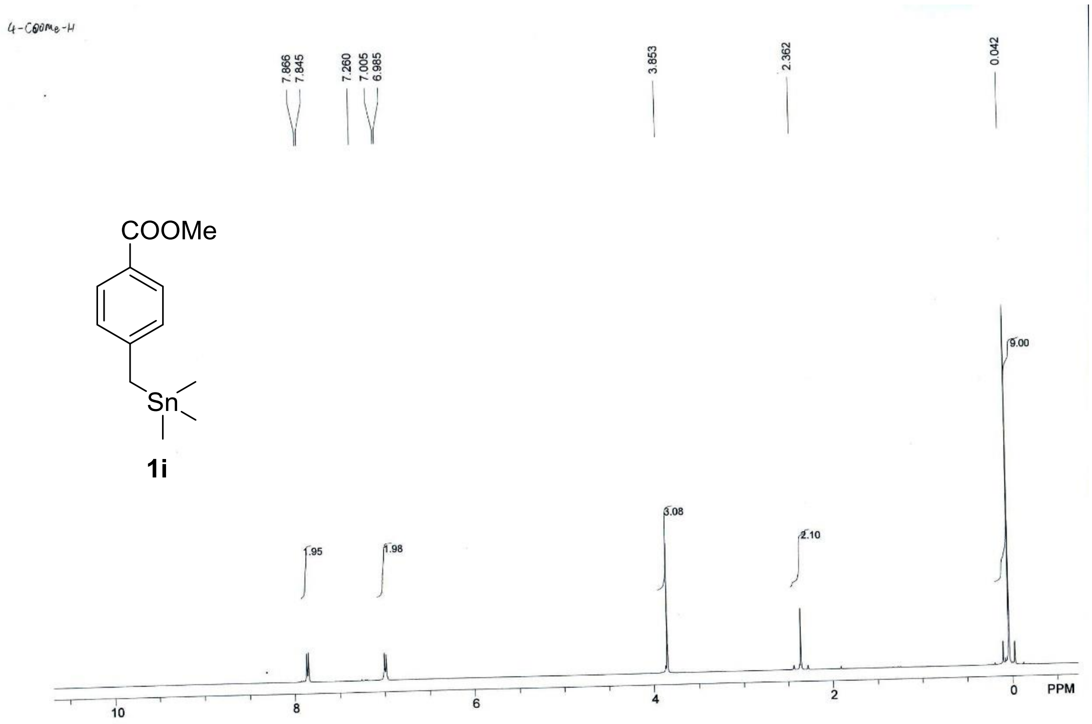
4-COOMe-H
7.866
7.845
7.260
7.005
6.985
3.853
2.362
0.042
COOMe
Sn
1i
1.95
1.98
3.08
2.10
9.00
10
8
6
4
2
0
PPM

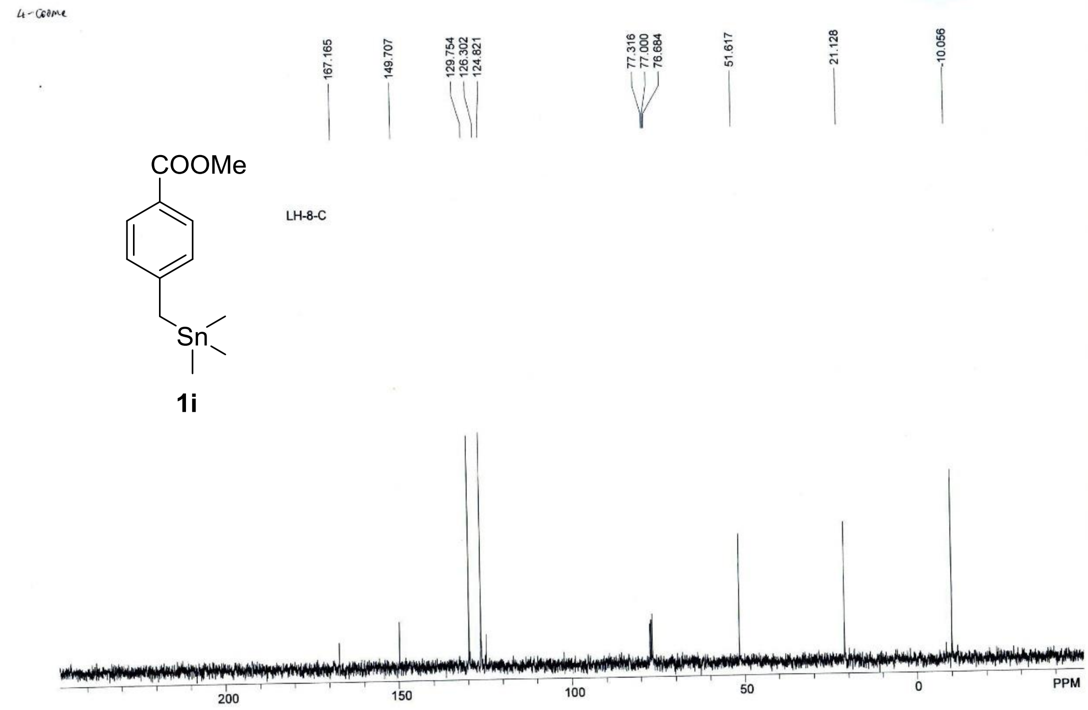
4-COOMe
167.165
149.707
129.754
126.302
124.821
77.316
77.000
76.684
51.617
21.128
-10.056
COOMe
LH-8-C
Sn
1i
200
150
100
50
0
PPM

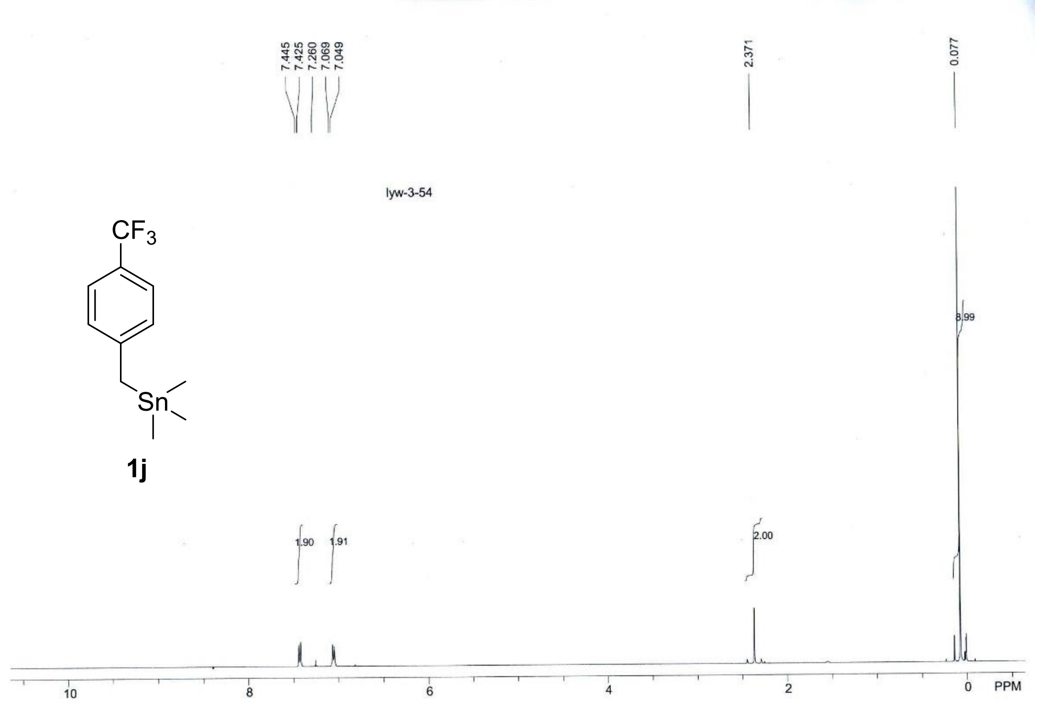
7.445
7.425
7.260
7.069
7.049
2.371
0.077
lyw-3-54
CF3
Sn
1j
8.99
1.90
1.91
2.00
10
8
6
4
2
0
PPM

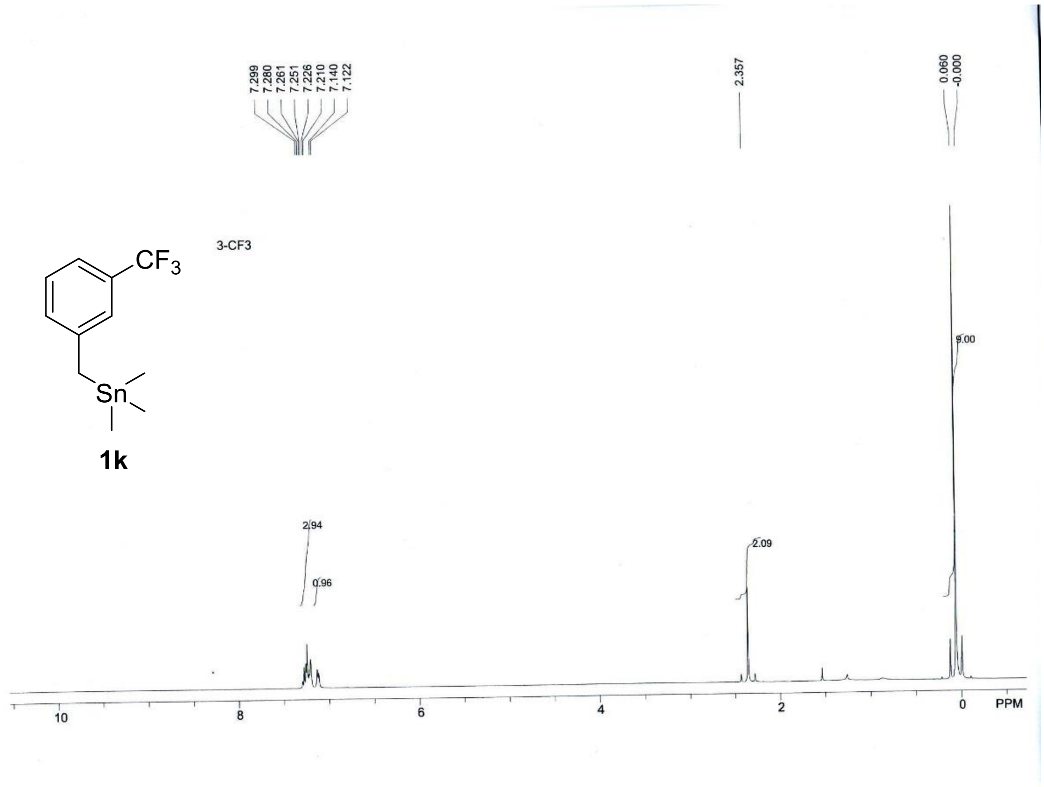
7.299
7.280
7.261
7.251
7.226
7.210
7.140
7.122
2.357
0.060
-0.000
3-CF3
CF3
Sn
1k
9.00
2.94
0.96
2.09
10
8
6
4
2
0
PPM

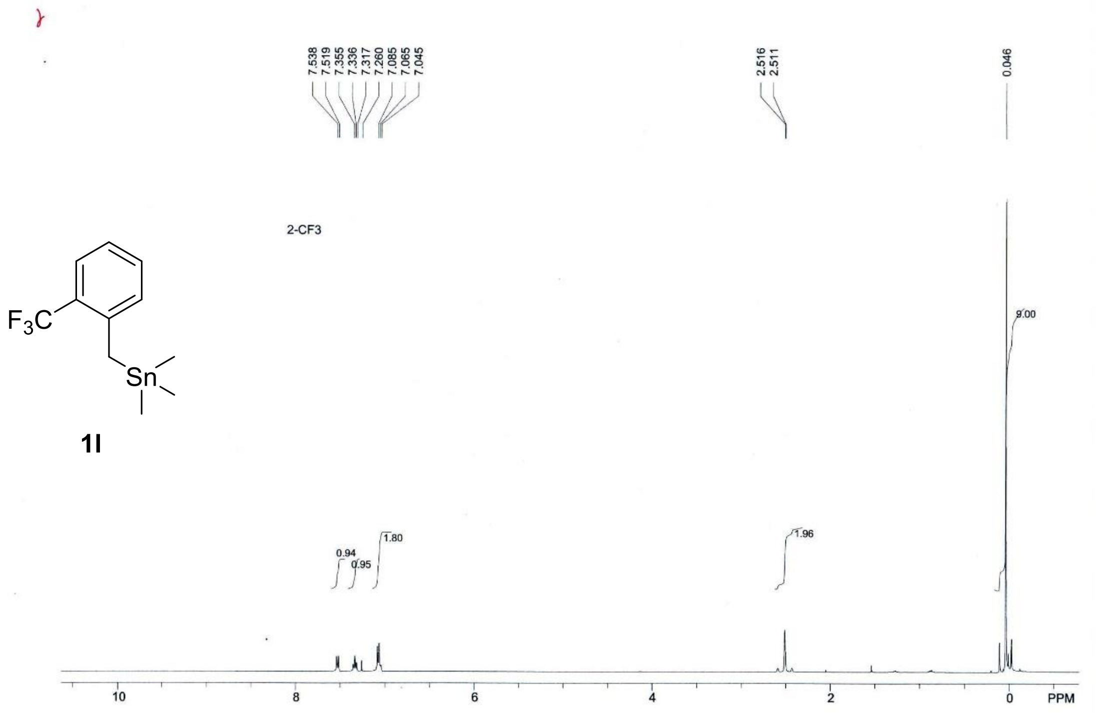
7.538
7.519
7.355
7.336
7.317
7.260
7.085
7.065
7.045
2.516
2.511
0.046
2-CF3
F_3C
Sn
1l
9.00
0.94
0.95
1.80
1.96
10
8
6
4
2
0
PPM

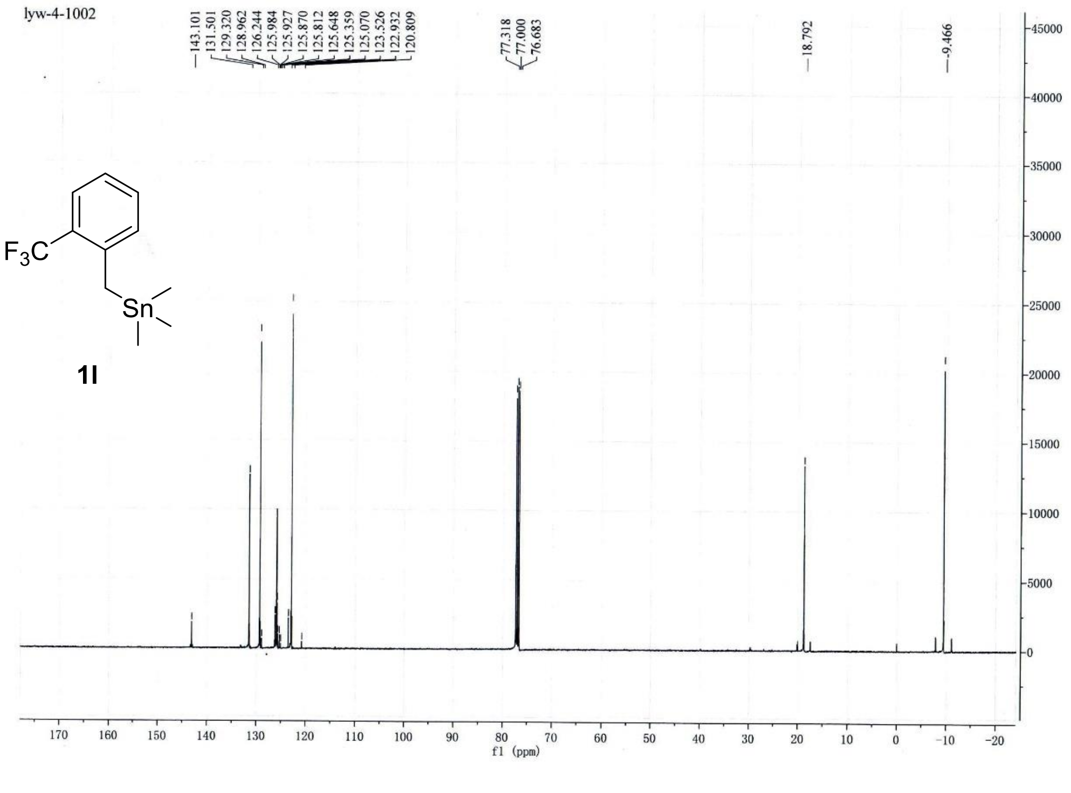
lyw-4-1002
143.101
131.501
129.320
128.962
126.244
125.984
125.927
125.870
125.812
125.648
125.359
125.070
123.526
122.932
120.809
77.318
77.000
76.683
18.792
-9.466
F_3C
Sn
1l
45000
40000
35000
30000
25000
20000
15000
10000
5000
0
170
160
150
140
130
120
110
100
90
80
70
60
50
40
30
20
10
0
-10
-20
f1 (ppm)

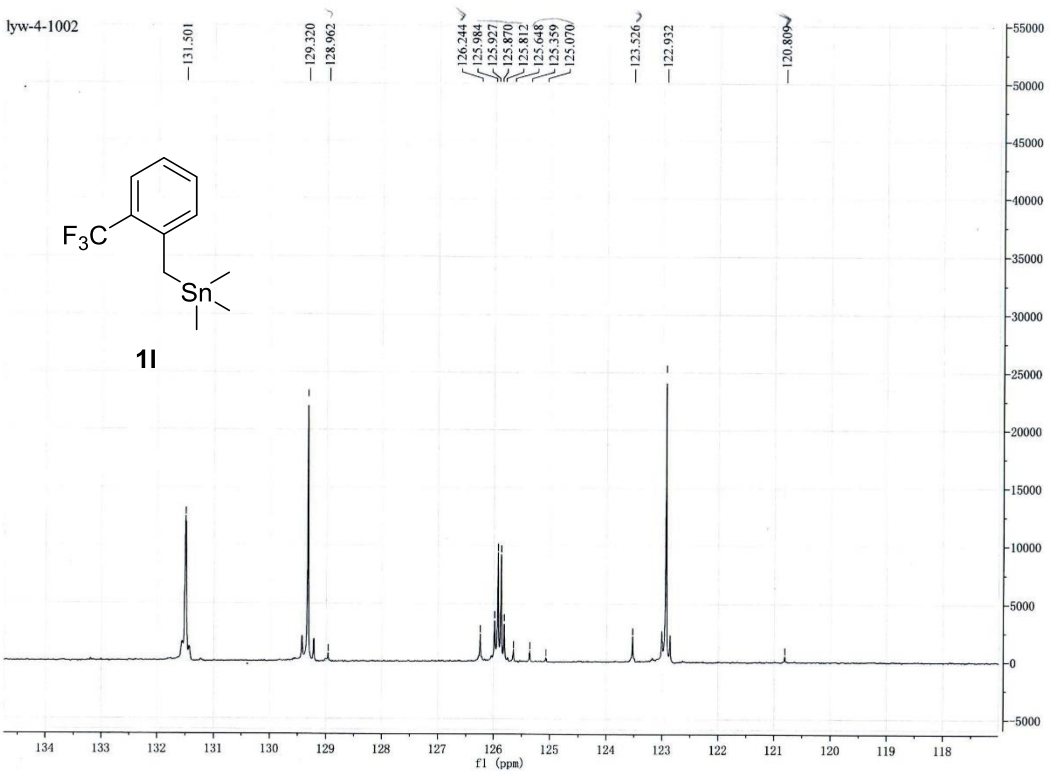

lyw-4-1002
131.501
129.320
128.962
126.244
125.984
125.927
125.870
125.812
125.648
125.359
125.070
123.526
122.932
120.809
F3C
Sn
1l
55000
50000
45000
40000
35000
30000
25000
20000
15000
10000
5000
0
-5000
134
133
132
131
130
129
128
127
126
125
124
123
122
121
120
119
118
f1 (ppm)

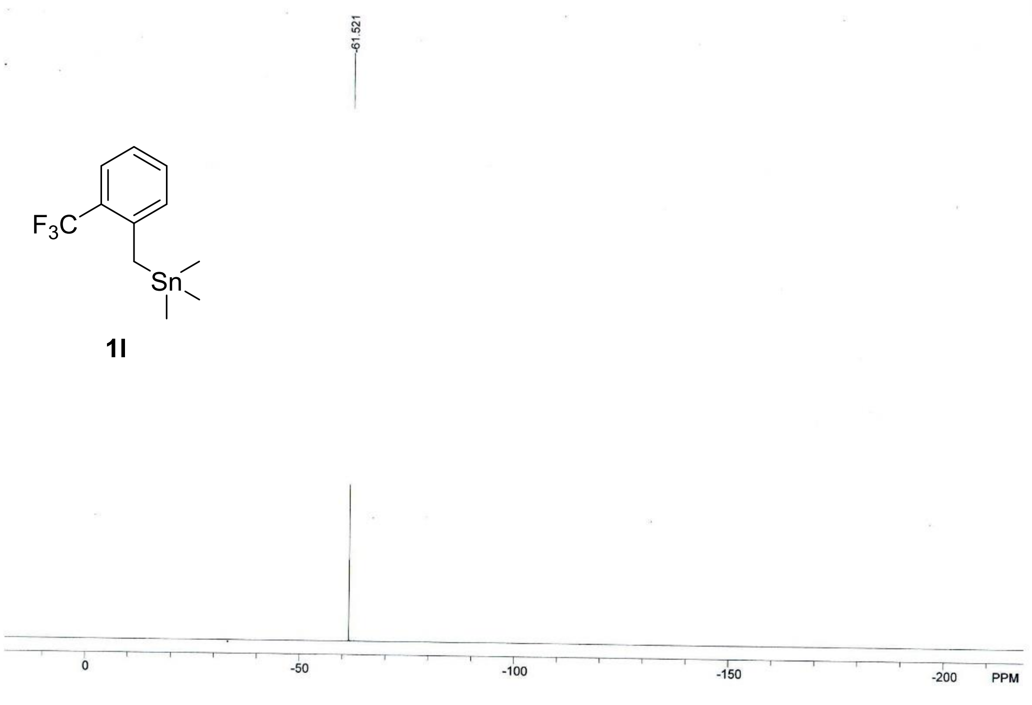

-61.521
F3C
Sn
1l
0
-50
-100
-150
-200
PPM

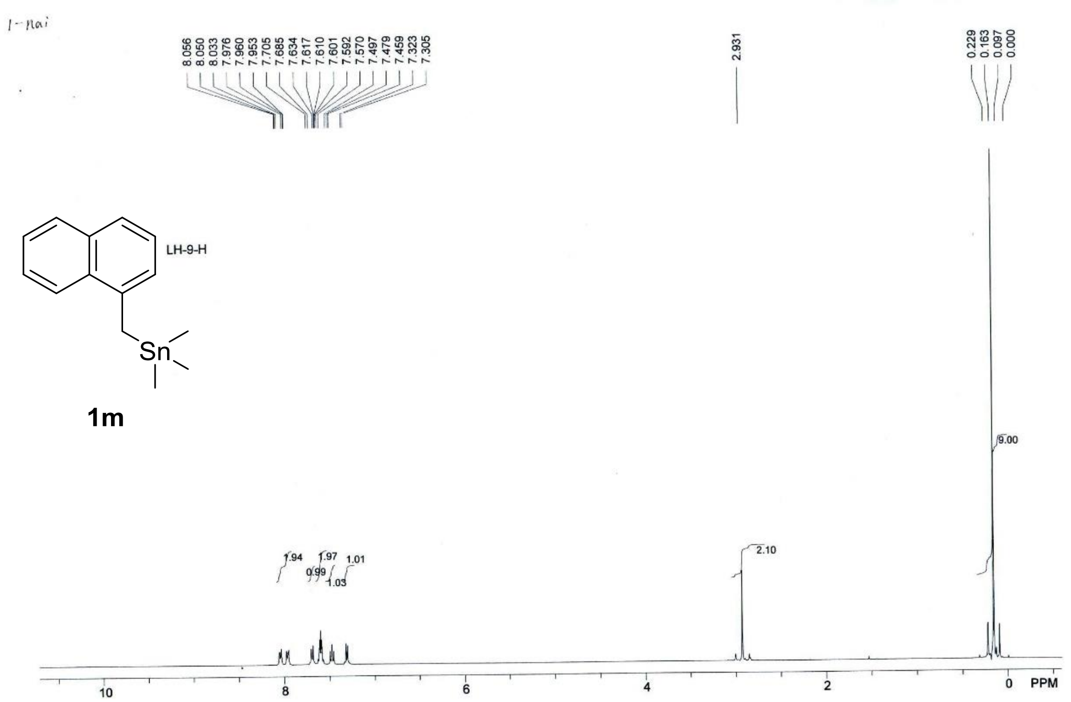
1-nai
8.056 8.050 8.033 7.976 7.960 7.953 7.705 7.685 7.634 7.617 7.610 7.601 7.592 7.570 7.497 7.479 7.459 7.323 7.305
2.931
0.229 0.163 0.097 0.000
LH-9-H
1m
9.00
2.10
1.94 0.99 1.97 1.03 1.01
10
8
6
4
2
0
PPM

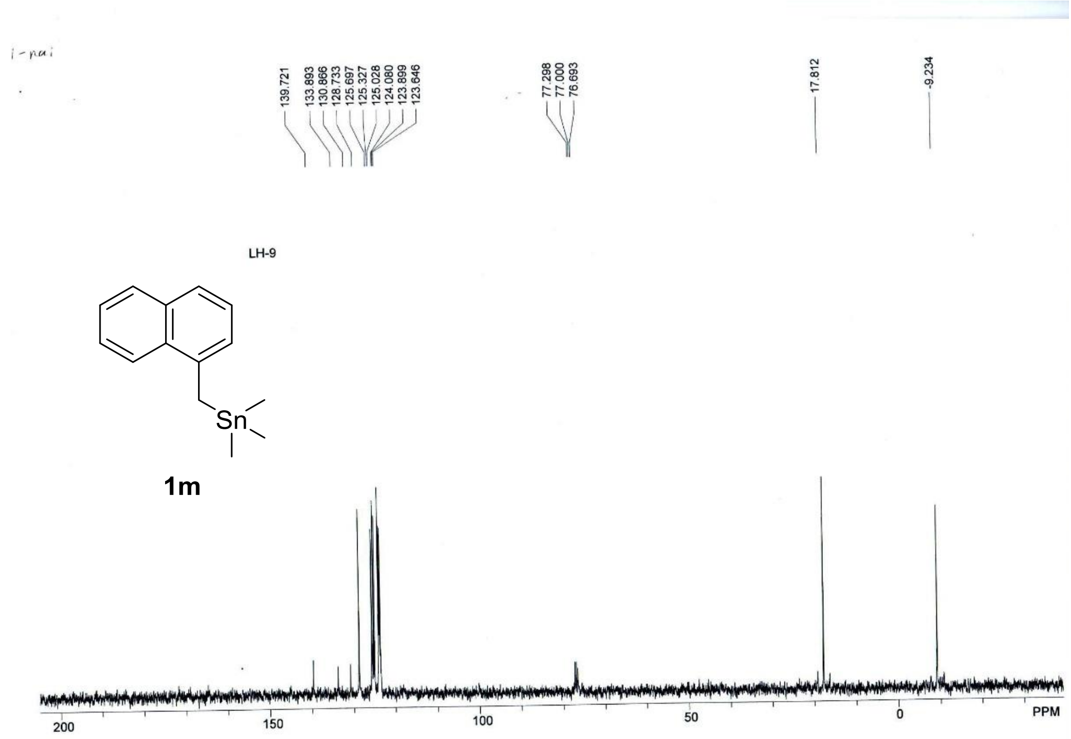
1-nai
139.721 133.893 130.866 128.733 125.697 125.327 125.028 124.080 123.899 123.646
77.298 77.000 76.693
17.812
-9.234
LH-9
1m
200
150
100
50
0
PPM

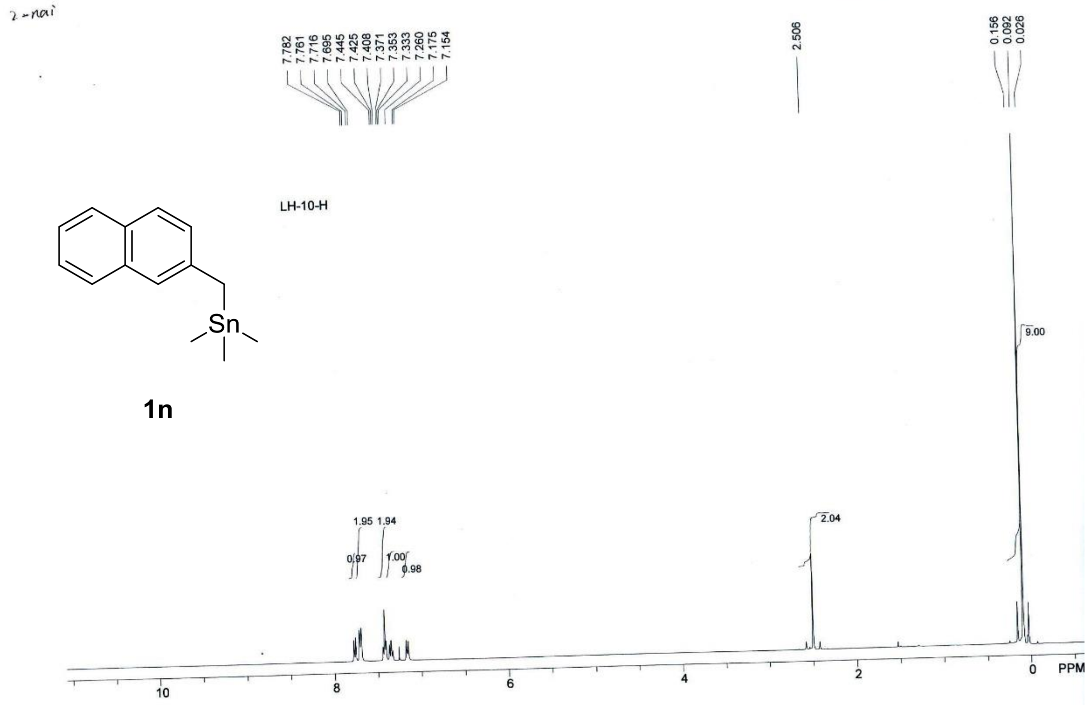

2-nai
7.782
7.761
7.716
7.695
7.445
7.425
7.408
7.371
7.353
7.333
7.260
7.175
7.154
2.506
0.156
0.092
0.026
LH-10-H
Sn
1n
9.00
1.95 1.94
0.97
1.00
0.98
2.04
10
8
6
4
2
0
PPM

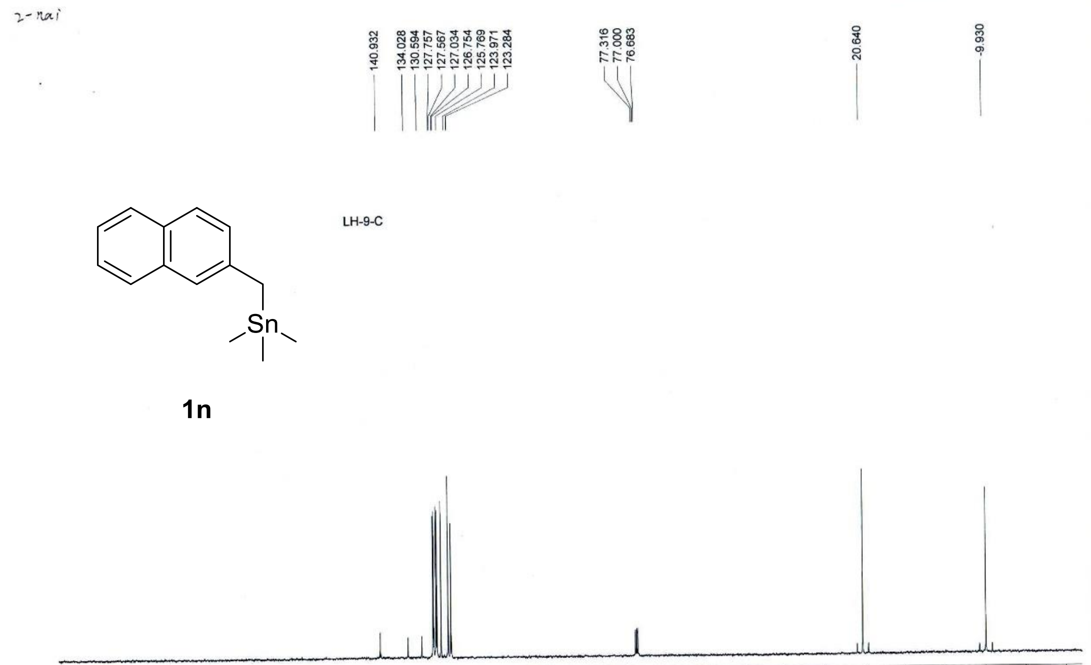

2-nai
140.932
134.028
130.594
127.757
127.567
127.034
126.754
125.769
123.971
123.284
77.316
77.000
76.683
20.640
-9.930
LH-9-C
Sn
1n
200
150
100
50
0
PPM

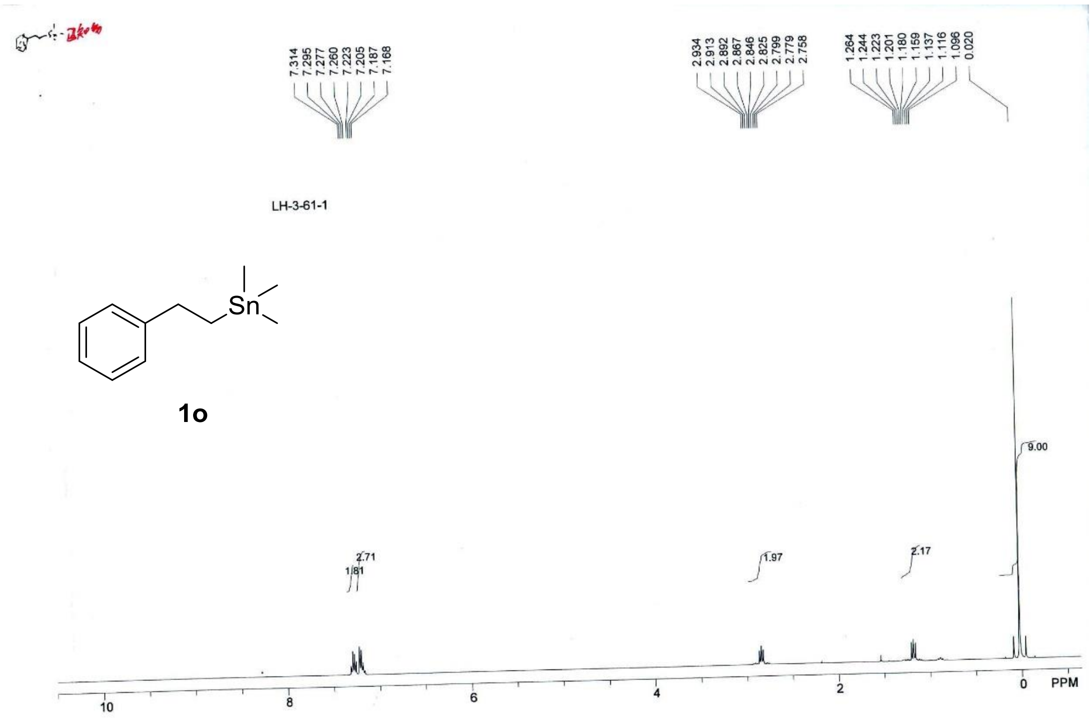
7.314
7.295
7.277
7.260
7.223
7.205
7.187
7.168
2.934
2.913
2.892
2.867
2.846
2.825
2.799
2.779
2.758
1.264
1.244
1.223
1.201
1.180
1.159
1.137
1.116
1.096
0.020
LH-3-61-1
Sn
9.00
2.71
1.81
1.97
2.17
10
8
6
4
2
0
PPM

1o

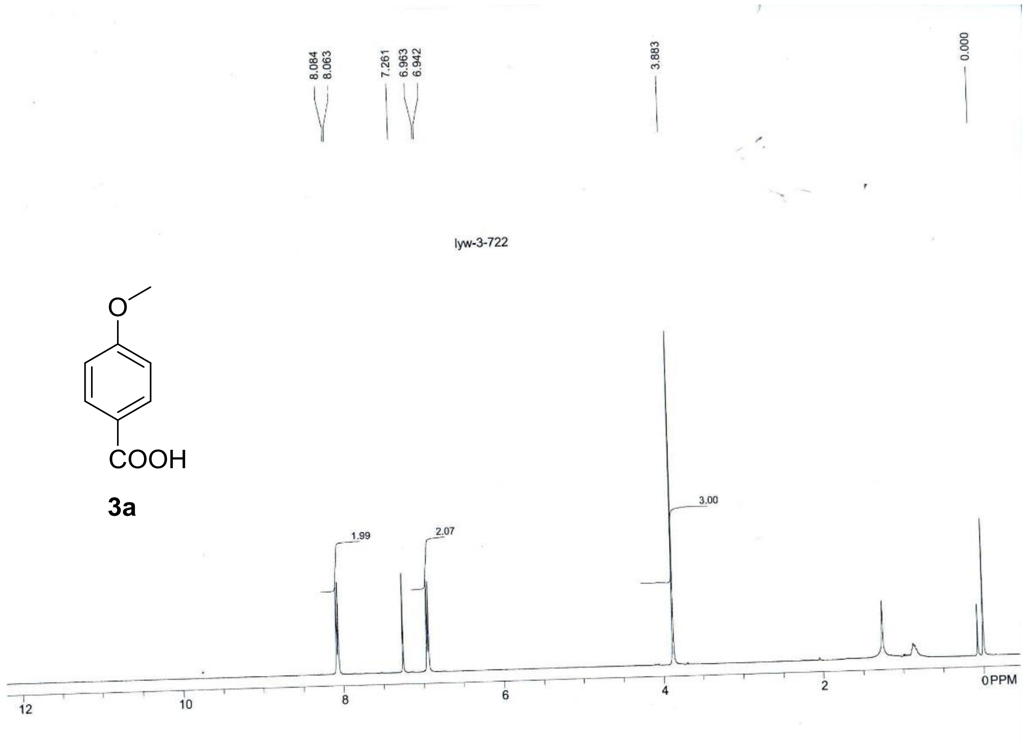
8.084
8.063
7.261
6.963
6.942
3.883
0.000
lyw-3-722
O
COOH
3.00
1.99
2.07
12
10
8
6
4
2
0PPM

3a

3b

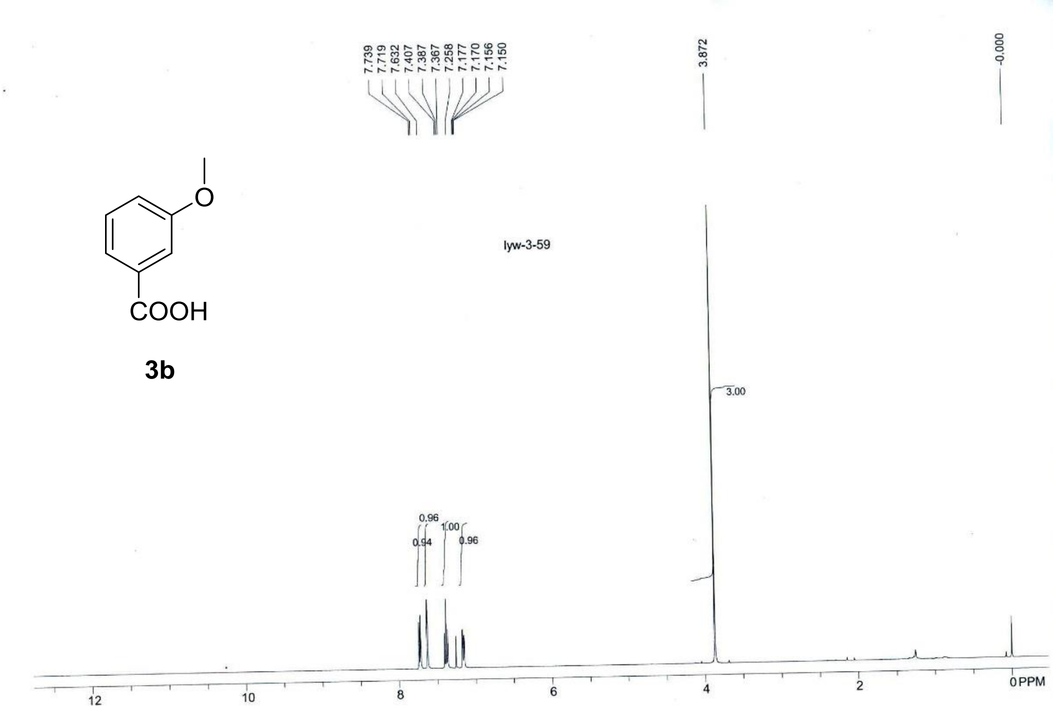
O
COOH
7.739
7.719
7.632
7.407
7.387
7.367
7.258
7.177
7.170
7.156
7.150
3.872
-0.000
lyw-3-59
3.00
0.96
0.94
1.00
0.96
12
10
8
6
4
2
0 PPM

3c

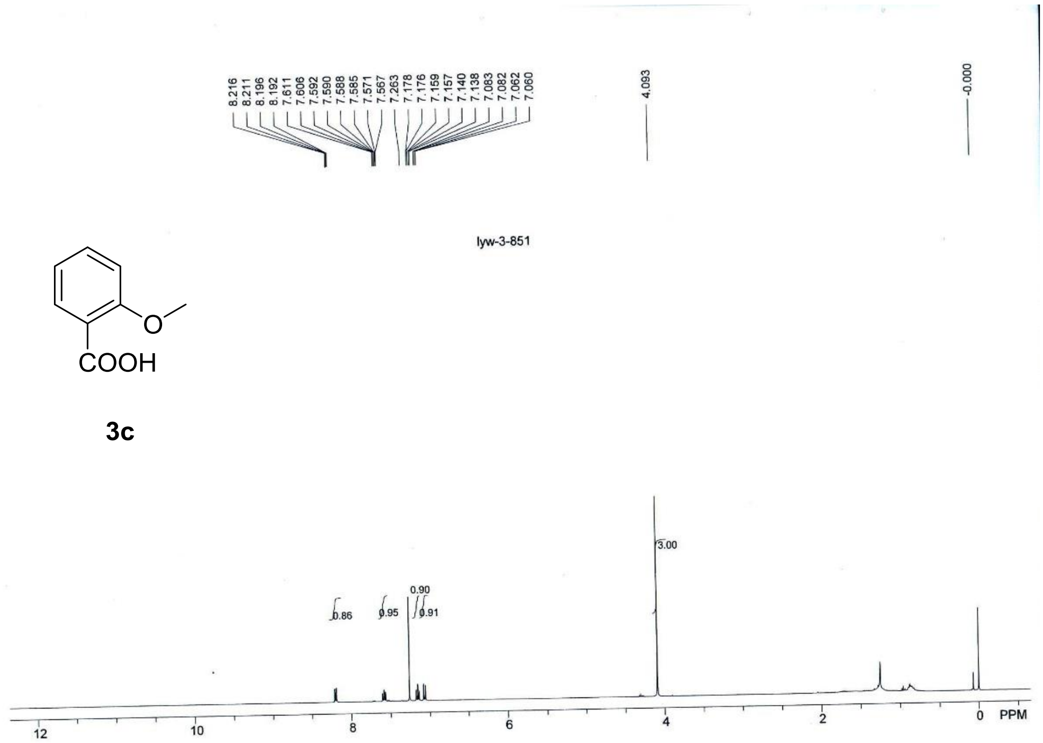
O
COOH
8.216
8.211
8.196
8.192
7.611
7.606
7.592
7.590
7.588
7.585
7.571
7.567
7.263
7.178
7.176
7.159
7.157
7.140
7.138
7.083
7.082
7.062
7.060
4.093
-0.000
lyw-3-851
3.00
0.90
0.86
0.95
0.91
12
10
8
6
4
2
0 PPM

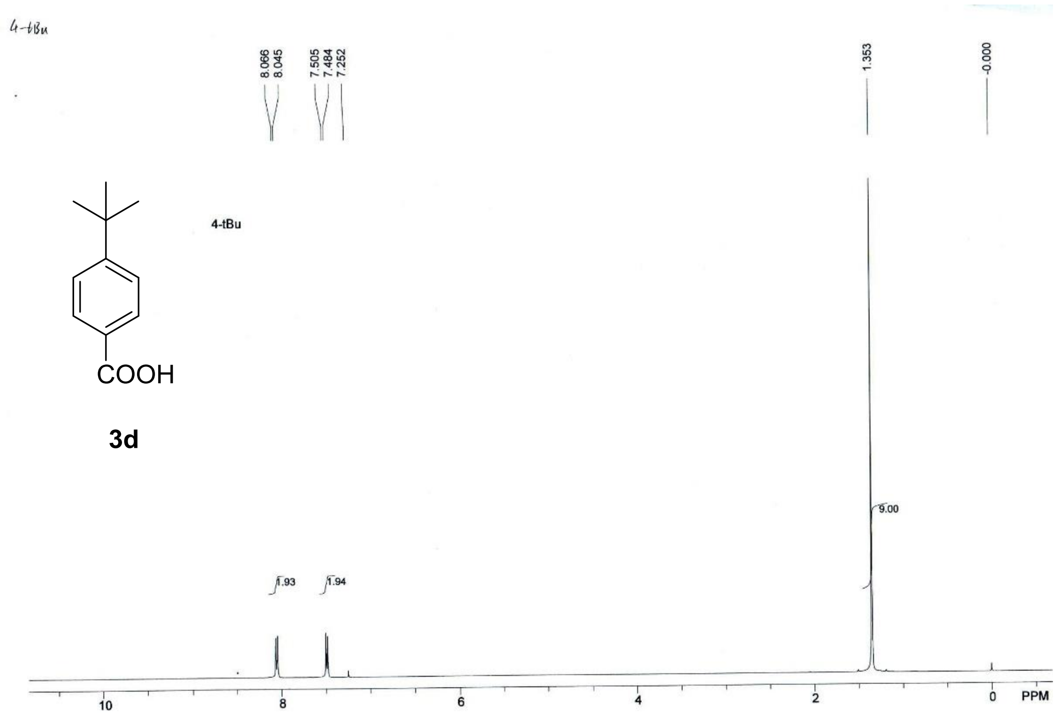
4-tBu
8.066
8.045
7.505
7.484
7.252
1.353
-0.000
4-tBu
COOH
3d
9.00
1.93
1.94
10
8
6
4
2
0
PPM

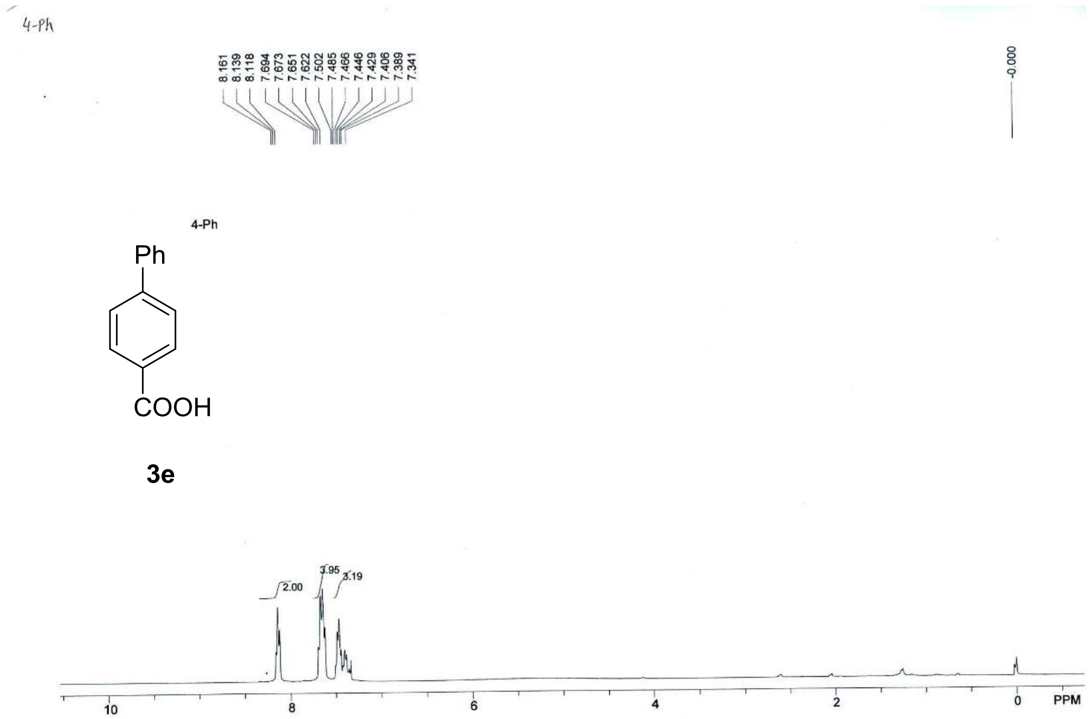
4-Ph
8.161
8.139
8.118
7.694
7.673
7.651
7.622
7.502
7.485
7.466
7.446
7.429
7.406
7.389
7.341
-0.000
4-Ph
Ph
COOH
3e
2.00
3.95
3.19
10
8
6
4
2
0
PPM

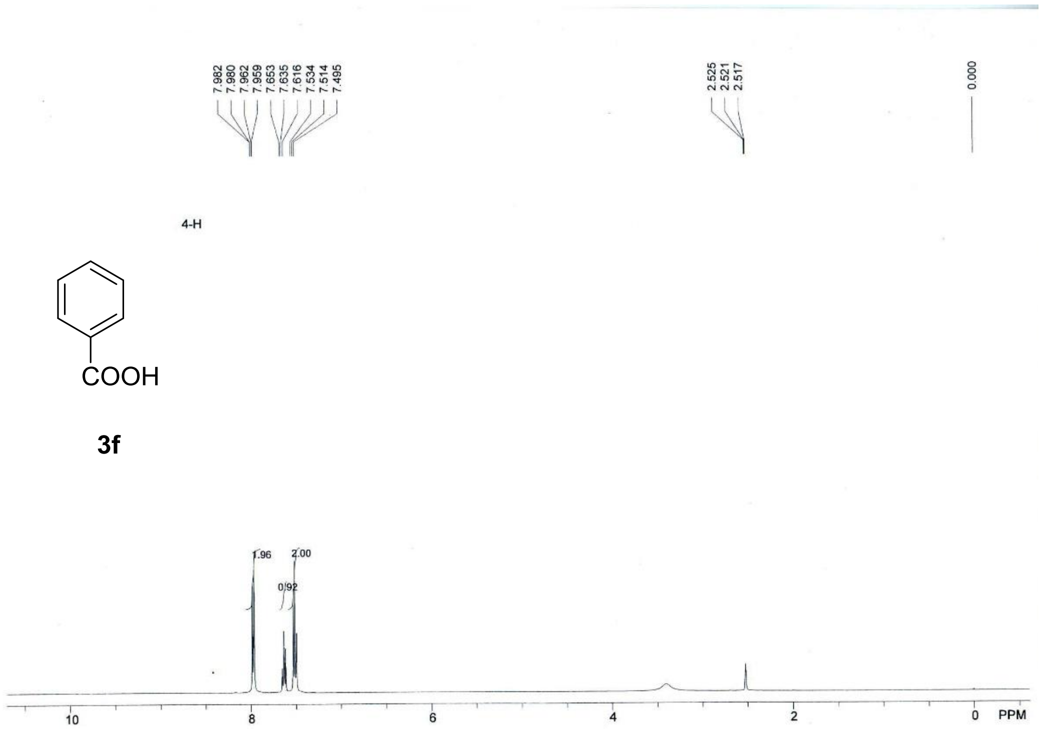
7.982
7.980
7.962
7.959
7.653
7.635
7.616
7.534
7.514
7.495
2.525
2.521
2.517
0.000
4-H
COOH
1.96
0.92
2.00
10
8
6
4
2
0 PPM

3f

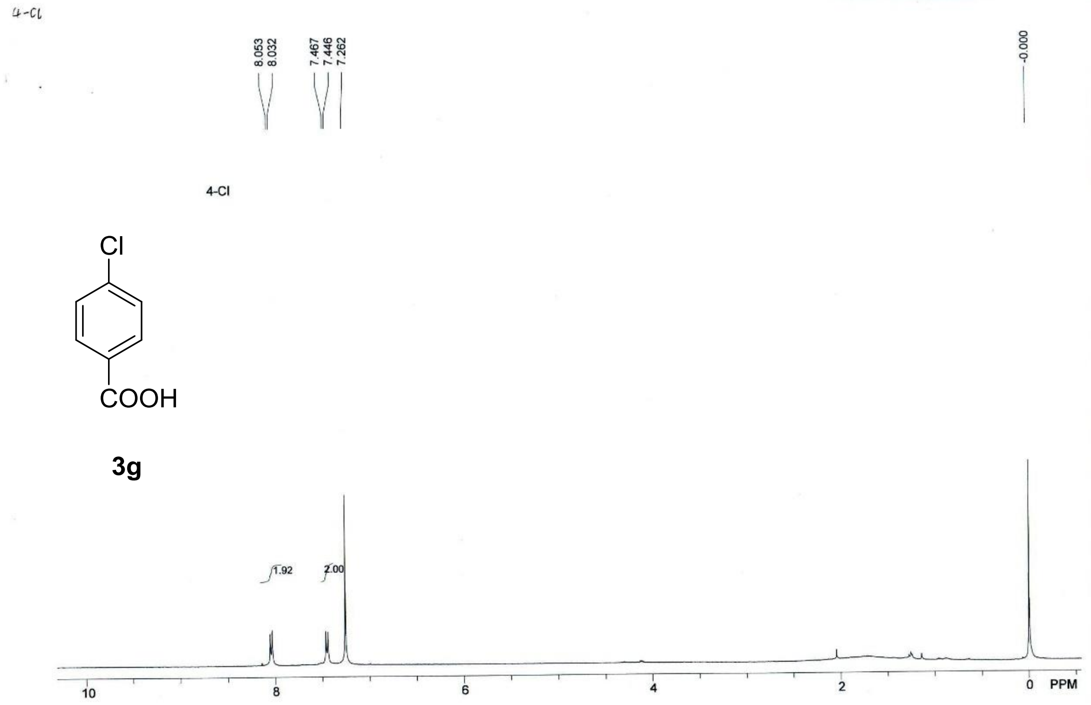
4-Cl
8.053
8.032
7.467
7.446
7.262
-0.000
4-Cl
Cl
COOH
1.92
2.00
10
8
6
4
2
0 PPM

3g

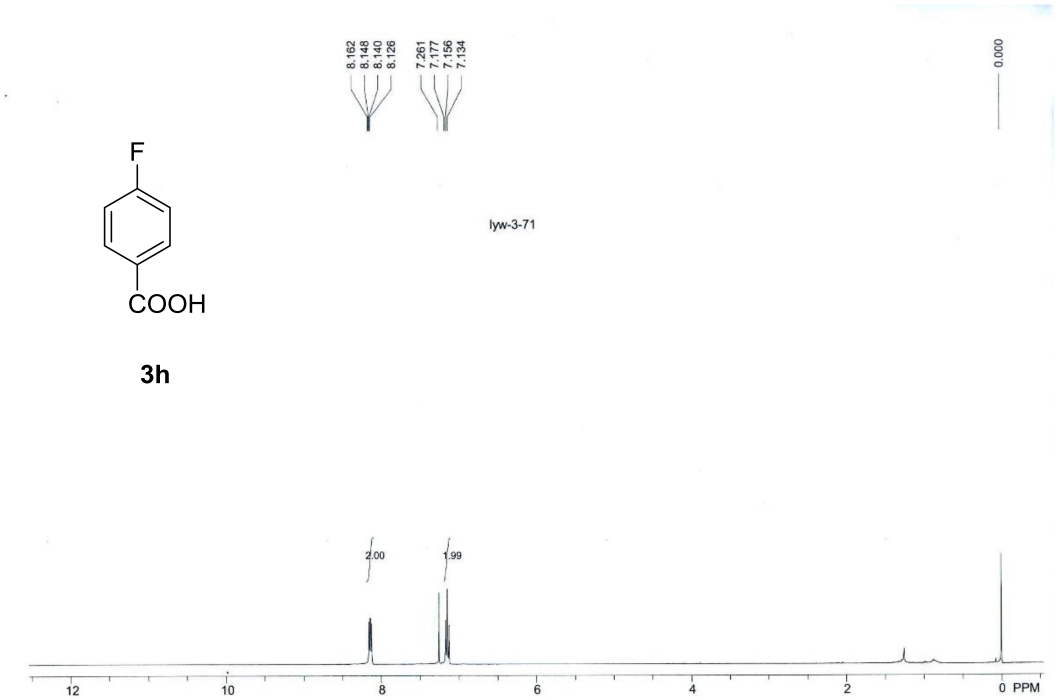
8.162
8.148
8.140
8.126
7.261
7.177
7.156
7.134
0.000
lyw-3-71
F
COOH
3h
2.00
1.99
12
10
8
6
4
2
0 PPM

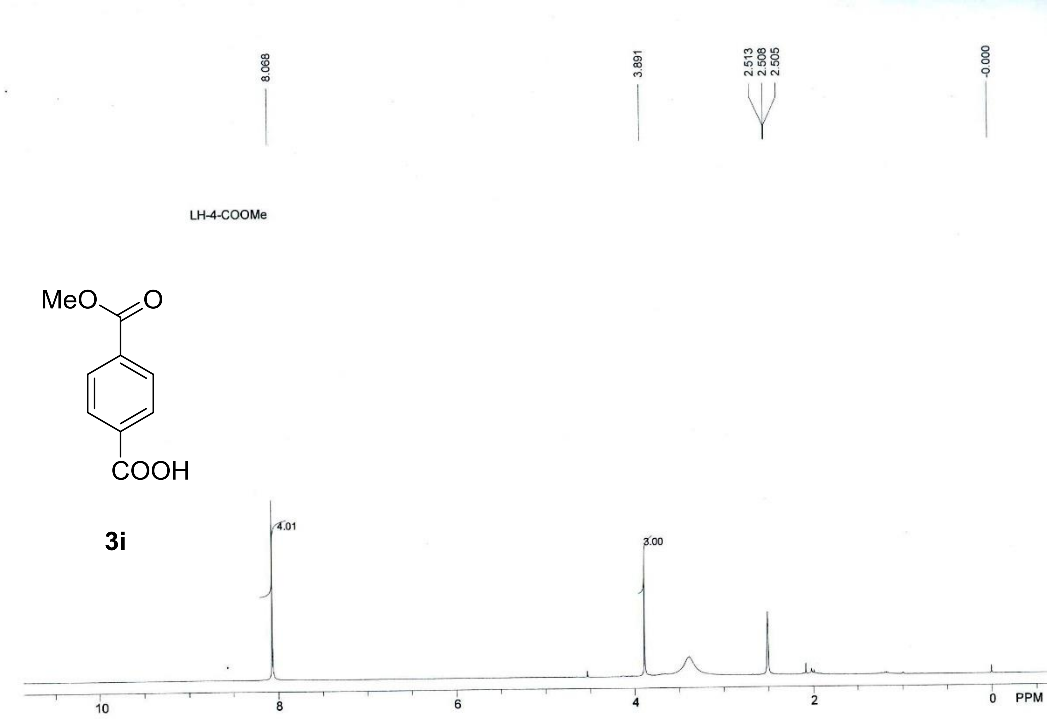
8.068
3.891
2.513
2.508
2.505
-0.000
LH-4-COOMe
MeO
O
COOH
3i
4.01
3.00
10
8
6
4
2
0 PPM

4-CF3

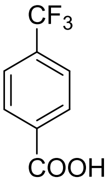

3j

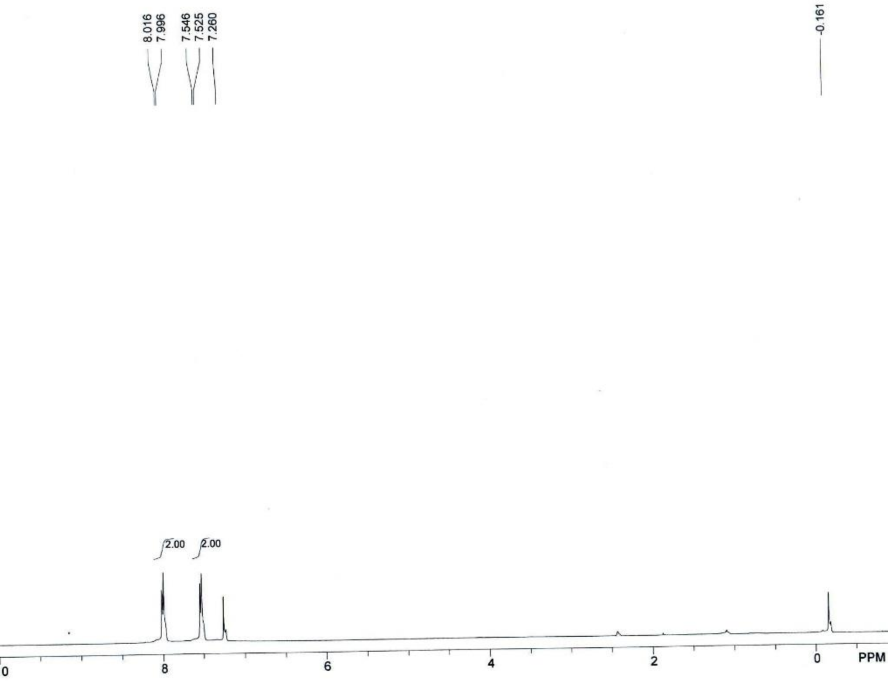

3-CF3-COON

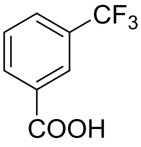

3k

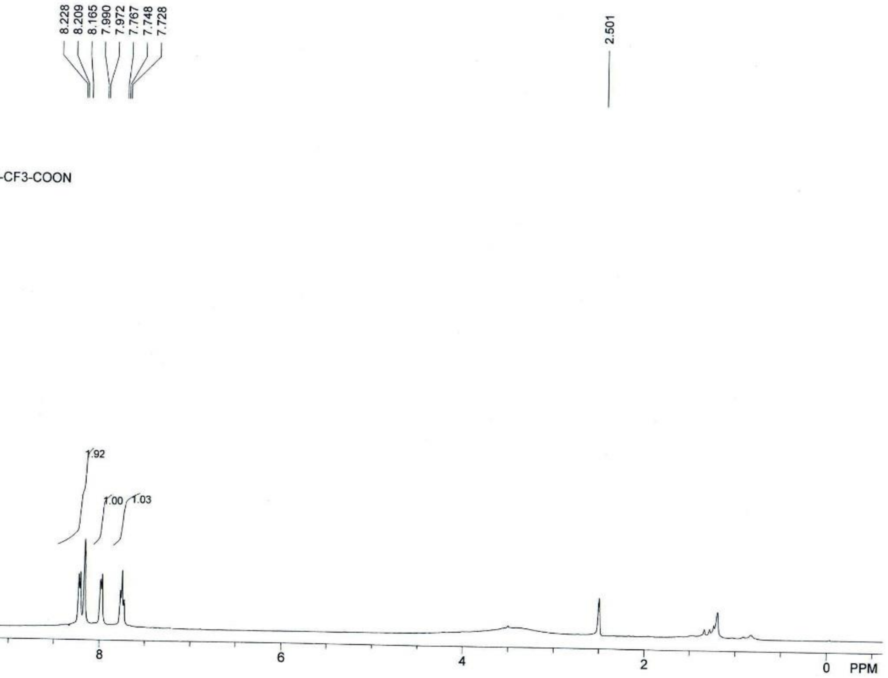

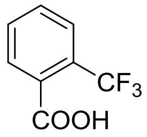

3l

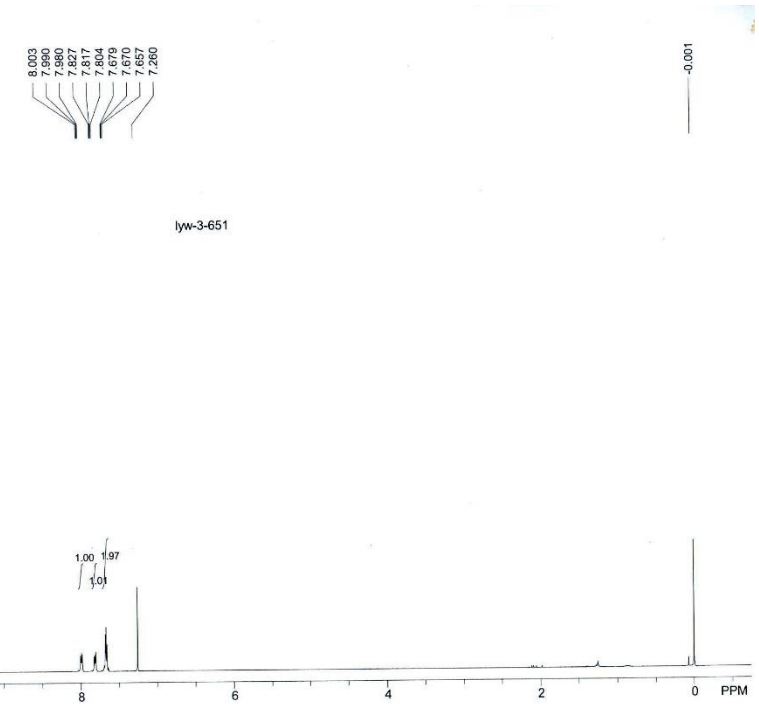
8.003
7.990
7.980
7.827
7.817
7.804
7.679
7.670
7.657
7.260
-0.001
lyw-3-651
1.00
1.01
1.97
12
10
8
6
4
2
0
PPM

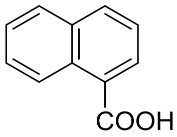

3m

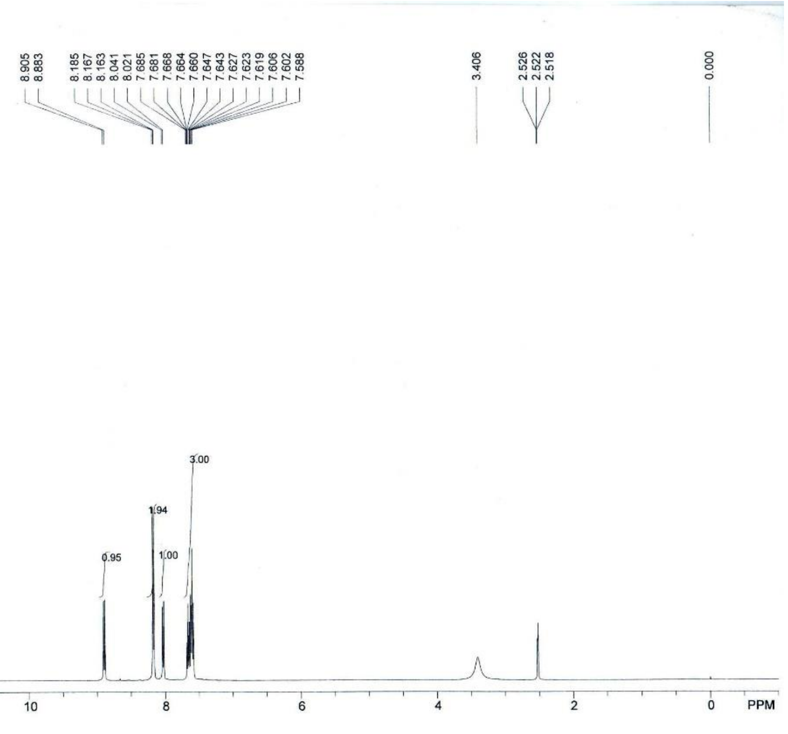
8.905
8.883
8.185
8.167
8.163
8.041
8.021
7.685
7.681
7.668
7.664
7.660
7.647
7.643
7.627
7.623
7.619
7.606
7.602
7.588
3.406
2.526
2.522
2.518
0.000
0.72
0.95
1.94
1.00
3.00
12
10
8
6
4
2
0
PPM

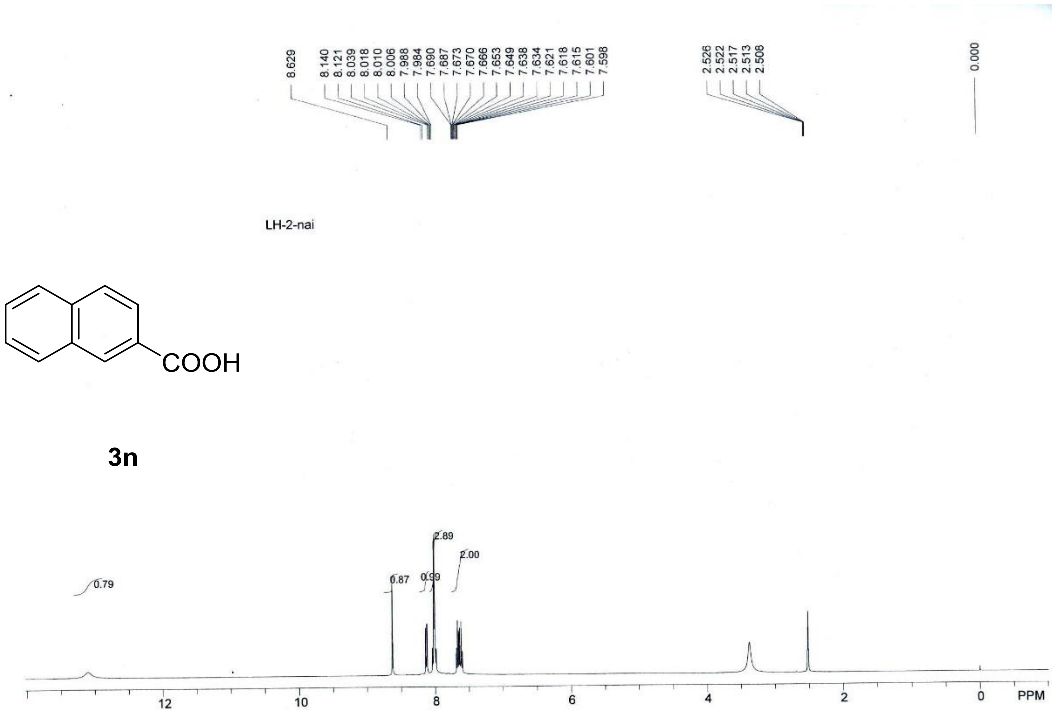

3n

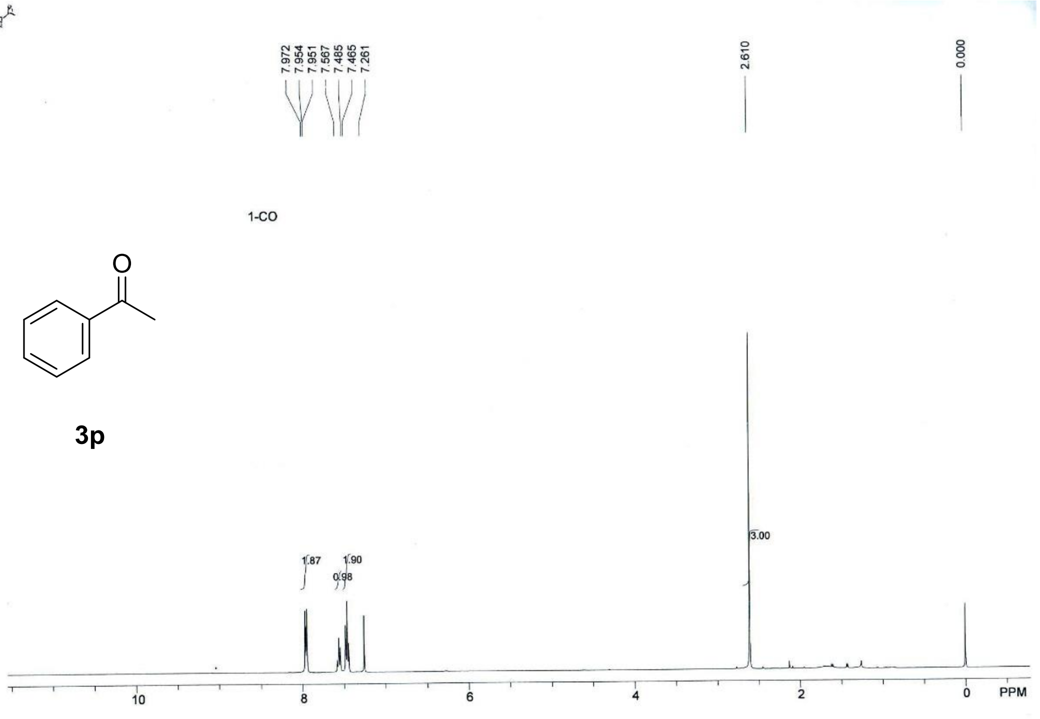

3p

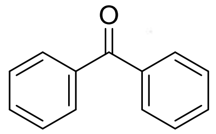

3q

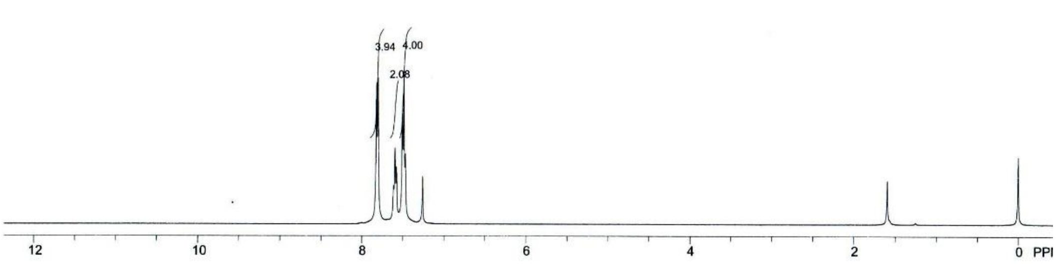
7.818
7.800
7.612
7.595
7.577
7.506
7.487
7.469
7.260
-0.000
lyw-3-871
3.94
4.00
2.08
12
10
8
6
4
2
0 PPI

References

1. Jousseaume, B. J., Duboudin, G. & Petraud, M. Synthese et etude par RMN ^{119}Sn de nouveaux composes benzyliques organostanniques. *J. Organomet. Chem.* **238**, 171-175 (1982).
2. Kitching, W. & Drew, G. σ-π Conjugation in benzyl derivatives of tin and mercury as probed by ^{119}Sn and ^{199}Hg resonance. *J. Org. Chem.* **46**, 2252-2260 (1981).
3. Moore, C. J. & Kitching, W. Sulfur dioxide insertion into benzyltrimethylstananes: kinetics and mechanism. *J. Organomet. Chem.* **59**, 225-230 (1973).
4. Mitchell, T. N. & Belt, H. J. Stannylated phosphines I. Preparation of (β-trimethylstannyl)-alkyldiphenylphosphines. *J. Organomet. Chem.* **368**, 167-172 (1989).
5. Sun, J., Wang, Y., Han, L. Q., Xu, D. W., Chen, Y. Y., Peng, X. H. & Guo, H. Photoinduced HBr-catalyzed C–Si bond cleavage of benzylsilanes and their subsequent oxidation into benzoic acids with air as the terminal oxidant. *Org. Chem. Front.* **1**, 1201-1204 (2014).
6. Correa, A. & Mart ń, R. Palladium-catalyzed direct carboxylation of aryl bromides with carbon dioxide. *J. Am. Chem. Soc*. **131**, 15974-15875 (2009).
7. Bonvin, Y., Callens, E. Larrosa, I., Henderson, D., Oldham, A. J., Burton,A. & Barrett, J. A. G. M. Bismuth-catalyzed benzylic oxidations with *tert*-butyl hydroperoxide**.** *Org. Lett.* **7**, 4549-4552 (2005).
8. Lamani, M. & Prabhu, K. R. An Efficient oxidation of primary azides catalyzed by copper iodide: a convenient method for the synthesis of nitriles**.** *Angew. Chem. Int. Ed.* **49**, 6622-6625 (2010).
